# Fenugreek Stimulates the Expression of Genes Involved in Milk Synthesis and Milk Flow through Modulation of Insulin/GH/IGF-1 Axis and Oxytocin Secretion

**DOI:** 10.3390/genes11101208

**Published:** 2020-10-16

**Authors:** Thomas Sevrin, Clair-Yves Boquien, Alexis Gandon, Isabelle Grit, Pierre de Coppet, Dominique Darmaun, Marie-Cécile Alexandre-Gouabau

**Affiliations:** 1 FRANCE Bébé Nutrition (FBN) Laboratory, 53000 Laval, France; thomas.sevrin@etu.univ-nantes.fr; 2 Mixed Research Unit 1280 Pathophysiology of Nutritional adaptations (UMR 1280 PhAN) Nantes University, Research Center in Human Nutrition-West (CRNH-O), Institute of Digestive Tract Diseases (IMAD), French National Research Institute for Agriculture, Food and Environment (INRAE), F-44000 Nantes, France; clair-yves.boquien@univ-nantes.fr (C.-Y.B.); alexis.gandon@univ-nantes.fr (A.G.); isabelle.grit@univ-nantes.fr (I.G.); Pierre.De-Coppet@univ-nantes.fr (P.d.C.); dominique.darmaun@univ-nantes.fr (D.D.); 3 Nantes University Hospital (CHU) Nantes, F-44000 Nantes, France

**Keywords:** fenugreek, galactagogue, milk synthesis, gene expression, lactating mammary gland, pituitary gland, insulin, oxytocin

## Abstract

We previously demonstrated galactagogue effect of fenugreek in a rat model of lactation challenge, foreshadowing its use in women’s breastfeeding management. To assess longitudinal molecular mechanisms involved in milk synthesis/secretion in dams submitted to fenugreek supplementation, inguinal mammary, pituitary glands and plasma were isolated in forty-three rats nursing large 12 pups-litters and assigned to either a control (CTL) or a fenugreek-supplemented (FEN) diet during lactation. RT-PCR were performed at days 12 and 18 of lactation (L12 and L18) and the first day of involution (Inv1) to measure the relative expression of genes related to both milk synthesis and its regulation in the mammary gland and lactogenic hormones in the pituitary gland. Plasma hormone concentrations were measured by ELISA. FEN diet induced 2- to 3-times higher fold change in relative expression of several genes related to macronutrient synthesis (*Fasn*, *Acaca*, *Fabp3*, *B4galt1*, *Lalba* and *Csn2*) and energy metabolism (*Cpt1a*, *Acads*) and in IGF-1 receptor in mammary gland, mainly at L12. Pituitary oxytocin expression and plasma insulin concentration (+77.1%) were also significantly increased. Altogether, these findings suggest fenugreek might extend duration of peak milk synthesis through modulation of the insulin/GH/IGF-1 axis and increase milk ejection by activation of oxytocin secretion.

## 1. Introduction

The World Health Organization (WHO) recommends exclusive breastfeeding for infants up to 6 months of age, based on evidence of its clear health benefits on mother–infant dyad [1,2,3]. Yet, 6 months after delivery, less than 40% of mothers are still breastfeeding in several high-income countries of North America and Europe [4]. Although the early cessation of breastfeeding is multifactorial [5], the perception that their milk secretion is insufficient to support adequate infant growth is reported by about 35% of lactating women [6,7]. Though perceived milk insufficiency due to psychological issues likely is more frequent than true insufficient milk secretion [5,8], the latter can result from many causes, ranging from true physiologic inability to lactate (5% of the cases) to suboptimal breastfeeding management [8,9]. It is well established that mothers’ milk production can often be increased through psychological support or maternal breastfeeding counseling [5,9]. Nevertheless, more and more healthy mothers try to enhance their milk supply by taking various nutritional supplements [10] presumed to have a galactagogue effect.

In the absence of a reference galactagogue molecule, the use of plant extracts has grown rapidly [10], with nowadays 15% to 25% of mothers using herbal remedies to increase their milk supply in Western countries [11,12]. Fenugreek is the most consumed, accounting for 50% of the plant extracts taken during breastfeeding [11], although strong evidence of its effectiveness and safety is scarce [11,13]. In a recent study using the deuterium oxide enrichment method to accurately measure milk secretion in a model of rat nursing large litters [14], we found that dietary fenugreek at a dose of 1 g/kg body weight/day increased milk production by 16% and offspring growth by 11% without any evidence for adverse metabolic effect on either dams or offspring [15], demonstrating a promising potential of fenugreek in the treatment of breastfeeding deficiency. However, the mechanisms underlying its effect remain poorly understood [16] and deciphering the targets of fenugreek in the lactation process should allow initiating careful support to mothers with lactation difficulties.

Milk production is mainly achieved by complex interactions signals both from the endocrine pathway and the local mammary gland environment. Indeed, in response to nipple areola stimulation during suckling, a nervous message is sent to the hypothalamus that stimulates the secretion of both prolactin and oxytocin, the main milk secretory hormones, at the pituitary level. Prolactin stimulates the synthesis of main milk macronutrients by binding to its receptors located on the surface of lactocytes (milk secretory cells). Oxytocin stimulates milk ejection by inducing the contraction of myoepithelial cells surrounding mammary secretory alveoli [17,18]. Insulin also plays an essential role by stimulating mainly protein and lactose synthesis in the mammary gland [19]. Finally, growth hormone (GH) and its effector insulin-like growth factor 1 (IGF-1), and thyroid hormones, can stimulate milk production directly in the mammary gland or by redirecting the nutrient flow toward breast tissues [20,21,22].

Among the therapeutic properties of its numerous bioactive constituents [23,24,25], such as trigonelline or diosgenin, fenugreek may increase prolactin secretion [26] due to an estrogenic action, which decreases the secretion of dopamine (a prolactin secretion inhibitor) in the hypothalamus [26,27,28]. Fenugreek may also increase food consumption through inhibition of leptin secretion [29]. Some fenugreek constituents had also been shown to increase GH secretion by rat pituitary cells [30]. In addition, fenugreek is a well-known hypoglycemic agent [25] due to its capacity to increase insulin secretion by β-pancreatic cells [24]. Finally, fenugreek is rich in antioxidant compounds [24,25] that could sustain an optimal mammary gland function. We hypothesized that fenugreek may increase milk production through an effect on the regulation of lactation.

The objectives of the present study were (i) to determine whether fenugreek impacted genes responsible for the synthesis of milk constituents and milk production, particularly candidate genes present in mammary and pituitary glands and responsible for the regulation of lactogenesis and milk ejection, and (ii) to determine the time course of the observed effects on the candidate genes at 3 time points: mid-lactation, late lactation and early mammary gland involution.

## 2. Materials and Methods

### 2.1. Animal Experiment

#### 2.1.1. Housing and Diets

Pregnant Sprague-Dawley rats, purchased from Janvier Labs (Le Genest-Saint-Isle, France) at first gestational day (G1), were housed individually in cages with wood chips located on ventilated racks kept at a constant temperature of 22 ± 1 °C and at a relative humidity of 50% ± 3%. Cages were placed in a room with a fixed 12 h light–dark cycle (light from 7:00 a.m. to 7:00 p.m.). Pregnant rats had access to water and food ad libitum.

During gestation, every dam received a control diet (CTL) based on AIN-93G diet with 20 g protein per 100 g of food. At parturition (G21 = L0), dams received ad libitum either the control diet (CTL) alone or the CTL diet supplemented with dry water extract of fenugreek seeds (Plantex, Sainte-Geneviève-des-Bois, France) (FEN) at 1 g/kg body weight/day as previously described [15]. The diets were manufactured by the “Experimental Food Preparation Unit” (INRAE-UPAE, Jouy-en-Josas, France).

#### 2.1.2. Experimental Design

The experimental protocol was approved by the Animal Ethics committee of Pays de La Loire and registered under reference (protocol APAFIS 2018121018129789) (Angers, France, 10 December 2018). All animal procedures were carried out in accordance with current institutional guidelines on animal experimentation in the EU (directive 2010/63/EU), in France (French Veterinary Department—A44276) and the French Ministry of Research in compliance with the commonly-accepted “3Rs”.

**Figure 1:**
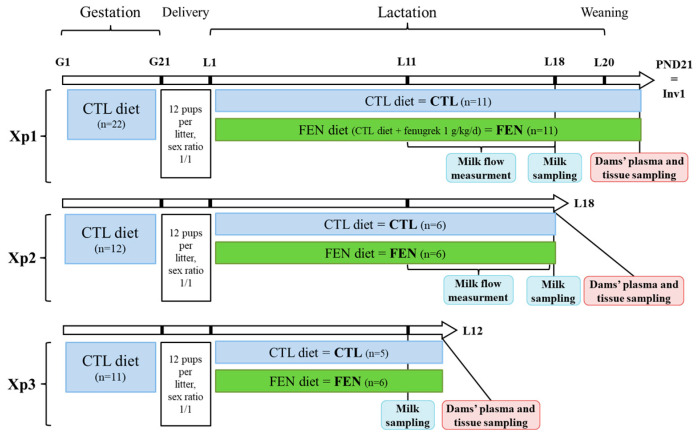
Experimental design: three series of animal experiments (Xp1, 2 and 3) were carried out, all with dams receiving either control (CTL) or fenugreek-supplemented diet (FEN), with different period of sampling for each experiment. G, L, PND and Inv correspond to the day of gestation, lactation, post-natal and involution days, respectively. Milk flow was measured by the deuterated water enrichment method [14,15] between L11 and L18. Tissues sampled were mammary and pituitary glands.

The study consisted of a succession of 3 separate experiments (Xp1, Xp2 and Xp3) with 22, 12 and 11 individual litters respectively, as shown in Figure 1. In the three experiments, all dams received CTL diet during gestation. Delivery occurred at G21 also considered as day 0 of lactation (L0) and dams were randomly assigned either to the CTL or FEN diet throughout lactation. Pups were pooled by sex and randomly adopted by CTL or FEN dams. Litters were homogenized to 12 pups with a sex ratio of 1/1 and balanced weight of 84 ± 2 g (Figure 1). The 3 experiments differed by the time at which animal follow up was stopped: post-natal day 21 for Xp1, L18 for Xp2 and L12 for Xp3. In Xp1 and Xp2, milk flow between dams and their pups was recorded between L11 and L18 using the deuterated water enrichment method and milk sample was collected at L18. In the Xp1, as previously described [14], pups were weaned at L20 to start the involution phase for dams’ mammary glands. At post-natal day 21, also assimilated to first involution day (Inv1), dams were fasted for 4 h before sacrifice and sampling of plasma and tissues. In Xp2, after milk sampling at L18, food and pups were removed from the cage for 2.5 h and 1 h respectively, before the beginning of dams’ sacrifice to avoid bias linked to hormone secretion during suckling. Finally, in Xp3, milk was sampled at L11 and dams were sacrificed at L12 after 1.5 h fasting and 1 h removal of pups (Figure 1).

#### 2.1.3. Dam-Litter Dyad Follow-Up and Milk Flow Measurement

Throughout lactation period, the dam weight, food and water consumption and total litter weight were recorded every two days from L0 to L11, and every day from L11 to L18. Pup weight was calculated as litter weight divided by litter size (12) and weight gain by subtracting weight al L0 from daily weight.

Milk flow was measured by the water turnover method as previously described [14,15]. Briefly, at L11, after 4%, isoflurane anesthesia, mothers received an injection of 4.92 ± 0.10 g/kg body weight D_2_O (99.9 mole% D_2_-enrichment, Sigma-Aldrich, Lyon, France) into a tail vein. Plasma samples were collected from dams by tail snip from L11 to L15 and pooled urine samples from their own whole litters were collected from L12 to L18 by stimulating lower belly with an iced cotton bud. The D_2_O enrichment of both plasma and urine samples was measured using the Fourier Transform infrared spectrophotometer α II^®^ (Brucker, Rheinstetten, Germany). Milk flow calculation was performed using a bi-compartmental model [15] as no significant intra-litter sexual dimorphism was observed in previous experiments [14]. Flow constants of the model (h^−1^) were then determined with the SAAM II^®^ software (The Epsilon Group, Charlottesville, Virginia, US) and the dams’ total body water (g) using the intercept of dams D_2_O concentration curve. The water flow, from dams to their own litter (g/h), was calculated as the product of total body water by flow constant from dam to the litter and was expressed in g/day by multiplying by 24.

#### 2.1.4. Milk, Plasma and Tissue Sampling

After 20 min dam/pups’ separation and intraperitoneal injection of oxytocin (1 unit of Syntocinon^®^; Sigma-Tau, Ivry-sur-Seine, France) to stimulate milk ejection, dams were anaesthetized with 4% of isoflurane, and milk was manually collected and stored at −20 °C until analysis. Blood samples were collected before animal sacrifice via intra-cardiac puncture and placed into Ethylene Diamine Tetraacetic Acid (EDTA) tubes (Pfizer-Centravet, Plancoët, France), centrifuged at 1132 g for 15 min at 4 °C, and plasma were stored at −20 °C until analysis. Dams were sacrificed by intra-cardiac injection of 0.5 mL Exagon^®^ (Richter pharma, Wels, Austria). Left inguinal mammary gland and pituitary gland were removed, sampled and immediately frozen in liquid nitrogen before storage at −80 °C.

### 2.2. Determination of Gene Expression in Mammary and Pituitary Glands Using RT-qPCR

#### 2.2.1. RNA Extraction and Purification

The entire pituitary gland (weight 19 ± 5 mg) or 28 ± 8 mg (mean ± standard deviation (SD)) of the inguinal mammary gland were placed into 2 mL Eppendorf tubes with glass beads in carbonic ice, and were then crushed using Precellys Evolution Homogenizer^®^ (Ozyme, Saint-Cyr l’Ecole, France) at 4000 rpm for 1 min. Total RNA was isolated from each tissue sample and purified following the NucleoSpin^®^ RNA/Protein (Marchery-Nagel, Düren, Germany) extraction/clean up protocol. RNA was eluted in 60 or 100 µL of RNase-free water. RNA concentration was measured at 260 nm and sample quality determined by ratios of 260/280 and 260/230 nm (Supplementary Table S1) using a NanoVue Plus^®^ (GE Healthcare, Little Chalfont, UK). As both ratios were in the range of 2.0–2.3, this guaranteed of sufficient pure RNA with low contamination by proteins and extraction solvents [31].

#### 2.2.2. Reverse Transcription (RT)

Complementary DNA (cDNA) was prepared from 1.5 µg of purified total RNA using SuperScript^®^ IV reverse transcriptase (Invitrogen, Villebon-Sur-Yvette, France) according to the manufacturer’s instructions. Moreover, some samples were randomly chosen to perform negative RT (RT^–^) by replacing SuperScript^®^ IV with water, to verify the absence of non-specific cDNA amplification in samples. Reverse transcription was performed with a SimplyAmp^®^ thermal cycler (Applied Biosystem, Villebon-Sur-Yvette, France) with thermal protocol of 23 °C for 10 min, 50 °C for 10 min and 80 °C for 10 min. Finally, the cDNA obtained were diluted to 1:40 before quantitative analysis.

#### 2.2.3. Primers Design and Validation

A total of 54 genes linked to milk constituents secretion and its regulation and lactogenic hormones secretion were studied [19,32,33] (Appendix A, Table A1). Primer pairs used for qPCR were designed based on the sequence obtained from the online gene section of National Center for Biotechnology Information (NCBI) (https://www.ncbi.nlm.nih.gov/gene/) and using open-source PerlPrimer v1.1.21 software (http://perlprimer.sourceforge.net.) [34] according to the following parameters: primer melting temperature ranging from 58 to 62 °C with a maximal difference of 2 °C, amplicon size ranging from 100 to 150 bp, primer length ranging from 20 to 24 bp, span intron/exon boundary and overlap intron/exon boundary by 7 bases to avoid the possibility of genomic DNA contamination. The selected primers had, if possible, no expandable dimer and a total dimerization with the lowest possible force (dG < 3). Then, to check for their selectivity and the absence of potential genomic DNA amplification, selected primers were blasted using Primer-BLAST of NCBI (https://www.ncbi.nlm.nih.gov/tools/primer-blast/index.cgi), against *Rattus norvegicus* “Refseq mRNA” and “Refseq representative genome”. Finally, all the primers were validated by measuring their efficiency (E) to amplify mammary gland or pituitary gland cDNA and by ensuring they did not amplify RT^–^. Efficiency was measured by preparing a pool of all samples of 1:40 dilution of cDNA which was then serially diluted 5 times to 1:2. The curve of cycle quantification value (Cq) function of log_2_(dilution) was linearly fitted and PCR efficiency (E) was calculated according to the following formula:

E (%) = (2^(−1/slope)^ − 1) × 100

Sample cDNA amplifications were compared to the five RT^–^ amplifications by calculating ΔCq_(RT-)_ = mean Cq_(RT-)_—mean Cq_(sample)_, setting a Cq value of 50 assigned in case of no amplification (Supplementary Table S1). All genes with their official symbol (NCBI), main functions, primer sequences, amplicon length and efficiency are listed in Appendix A Table A1. Overall, the mean amplicon size was 124.7 ± 28.6 (mean ± SD). For mammary and pituitary glands, efficiencies were greater than 90 and ΔCq_(RT-)_ greater than 10, indicating good amplification capacity and negligible genomic DNA amplification.

#### 2.2.4. Quantitative Polymerase Chain Reaction (qPCR) Analysis

The qPCR was executed in Hard-Shell^®^ 96-well PCR plates (Bio-Rad, Marne-la-Coquette, France) on a CFX connect^TM^ Real-Time PCR detection instrument (Bio-Rad). Reactions were performed with a total volume of 15 µL, including 5 µL of 1:40 dilution of cDNA, 250 nM of forward and reverse gene-specific primers and iTAQ Universal SYBR^®^ Green Supermix (Bio-Rad). The qPCR conditions were as follows: initial denaturation at 95 °C for 5 min followed by 40 cycles of 95 °C for 10 s, 60 °C for 30 s and 72 °C for 10 s. To check the specificity of PCR products, a melting curve was performed by increasing temperature from 65 to 95 °C with a 0.5 °C increment and 10 s per step.

Relative gene expression was calculated using the 2^−ΔΔCq^ method as previously described [35]. For mammary gland, arithmetic mean Cq of three housekeeping genes (HKG): *Actb*, *Uxt* and *Rps9*, was used to calculate ΔCq (Cq_Gene of interest_—Cq_Housekeeping genes_). *Actb* is a classically used HKG while *Uxt* and *Rps9* are known to be very stable genes in lactating mammary gland [36,37]. For pituitary gland, arithmetic mean Cq of *Actb*, *Vcp* and *Rps9* was used as HKG to calculate ΔCq. *Vcp* was shown to be very stable in the brain in an RNAseq study of the lab (unpublished data) and the Cq values of the three genes were strongly correlated (r from 0.68 to 0.81). ΔΔCq values (ΔCq_Target group_—ΔCq_Reference group_) were calculated using the CTL diet at L12 as a reference group to highlight the gene expression evolution over the lactation period. Values of −ΔΔCq were considered to be representative to the Log2(Fold change) assuming that mean primer efficiency was close to 100% and relatively similar between genes (CV of 4.7% and 5.9% in mammary and pituitary glands, respectively). Finally, fold change expression of the FEN diet was calculated for each lactation period with 2^−ΔΔCq^ formula using, for each day, the CTL diet as a reference group.

### 2.3. Enzyme-Linked Immunosorbent Assay (ELISA) of Lactaogenic Hormones in Dam Plasma

Insulin (Rat Insulin ELISA kit^®^, ALPCO, Salem, NH, USA), IGF-1 (ELISA kit for Insulin Like Growth factor 1, Cloud-Clone Corp., Katy, TX, USA), prolactin (Prolactin rat ELISA, Demeditec Diagnosis GmbH, Kiel, Germany) and leptin (Rat Leptin ELISA Kit, Merck Milliport, Burlington, MA, USA) were measured in dams’ plasma, following manufacturers’ instructions. Plasma was diluted to 1:10 for the prolactin assay and to 1:100 for the IGF-1 assay. Optical density was read using a microplate reader Varioskan Lux^®^ (ThermoFisher Scientific, Waltham, MA, USA).

### 2.4. Statistical Analysis

To study the global impact of fenugreek on genes responsible for milk synthesis and regulation in the mammary gland, multivariate analysis was performed, using SIMCA P^®^ version 15.02 (Umetrics AB, Kinnelon, NJ, USA). The unsupervised Principal Component Analysis (PCA) and the supervised Partial Least Squares Discriminant Analysis (PLS-DA) were used [38] to cluster the mammary gland samples based on the selected gene expression profiles (−ΔΔCq values). PCA reduced the large set of variables (43 genes) into two principal components (PCA 1 and 2). PLS-DA enhanced the separation between the dietary groups (CTL and FEN) by summarizing the data into a few latent variables that maximized covariance between the response (dietary group) and the predictors (gene expression). The performance of the PLS-DA models was evaluated by the proportion of the total variance of the variables explained by the model (R2(X)), the proportion of the total variance of the response variable (dietary group) explained by the model (R2(Y)), the prediction ability of the model reflected by both a 7-fold cross-validation of the data (Q2) and the reliability of the PLS-DA model based on an analysis of variance (ANOVA) assessment of the cross-validatory (CV) predictive residuals (*p*-value of CV-ANOVA) of the model. The corresponding loading plot was used to determine the genes most responsible for separation in the PLS-DA score plot. Based on the PLS-DA results, genes were plotted according to their importance in separating the dietary groups in PLS-DA score plot and each gene received a value called the variable of importance in the projection (VIP). The variables with the highest VIP values (above 1) were the most powerful group of discriminators involved in the separation of CTL and FEN groups. Finally, in order to highlight diet-specific effect on mammary gland gene expression, lactation time effect was masked by building new PCA and PLS-DA models on a global matrix of normalized variables obtained using mean centering and standardization of variables by lactation point.

For univariate analysis, parametric tests were favored to maximize statistical power, such as the Student’s test used for diet comparison. Their validity was admitted as the residual distribution of variables was not significantly different from normal distribution according to the Shapiro–Wilk test and variance was homogenous according to the Brown–Forsythe test. Relative genes’ expressions in tissues and plasma hormone concentrations were analyzed using two way ANOVA with diet and period factors followed by Tukey’s post-hoc test for period pairwise comparisons (3 levels) and Sidak’s post-hoc test for diet pairwise comparisons (2 levels), as suggested by Kim [39]. Values of −ΔΔCq were chosen for statistical analysis of gene expression because distribution of −ΔΔCq values, unlike that of 2^−ΔΔCq^ values, was similar to normal distribution that enabled the use of parametric tests [40,41]. To determine the strength of the link between two variables, Pearson’s correlation test was used. All univariate tests were performed with the GraphPad prism^®^ software (San Diego, CA, USA), version 6. In all tests, *p* < 0.05 was considered as statistically significant.

## 3. Results

### 3.1. Fenugreek Supplementation Enhances Lactation Performances in a Model of Rat Nursing a Large Litter

Lactation performance was measured in experiments 1 and 2 (Xp1 and Xp2). As no significant interaction effect was found between experiments and diet effects using an ANOVA, data from Xp1 and Xp2 were pooled. Dam water intake was similar in control diet (CTL) and fenugreek-supplemented diet (FEN) groups throughout the lactation period. Conversely, maternal food intake, pup final weight gain and milk flow from mother to pups all increased significantly, by 10.0%, 7.1% and 15.3%, respectively (Table 1).

### 3.2. Different Patterns of Gene Expression in the Mammary and Pituitary Glands and of Plasma Hormone Concentration throughout Lactation and Involution

Multivariate and univariate statistical analysis were performed to investigate the temporal pattern in gene expression in mammary gland from mid-lactation to the beginning of involution.

The genes with the highest expression in mammary gland were *Csn2*, *Wap*, *Mtco1* and *Lalba* with ΔCq ≤ −6 and codes for major milk proteins and actors of mitochondrial oxidative phosphorylation (Table 2 and Table 3). *Plin2* and *Fas*, two genes involved in lipid synthesis, were also strongly expressed in the mammary gland with ΔCq < −1. Conversely, *Cpt1a*, *Acads*, *Mtor*, *Igf1r Esr1*, *Insr*, *Lxra*, *Scd1*, *Pgm1* and *Dgat2* were the least expressed genes in mammary gland over the entire lactation period, with ΔCq > 4 (Table 2, Table 3, Table 4 and Table 5). In pituitary gland, *Prl* and *Gh1* were both very intensively expressed, with ΔCq < −6 highlighting the high level of secretion of prolactin and GH during lactation (Table 6). In contrast, *Oxt*, *Trhr* and *Vipr2* were the less expressed genes with ΔCq > 4.

**Figure 2:**
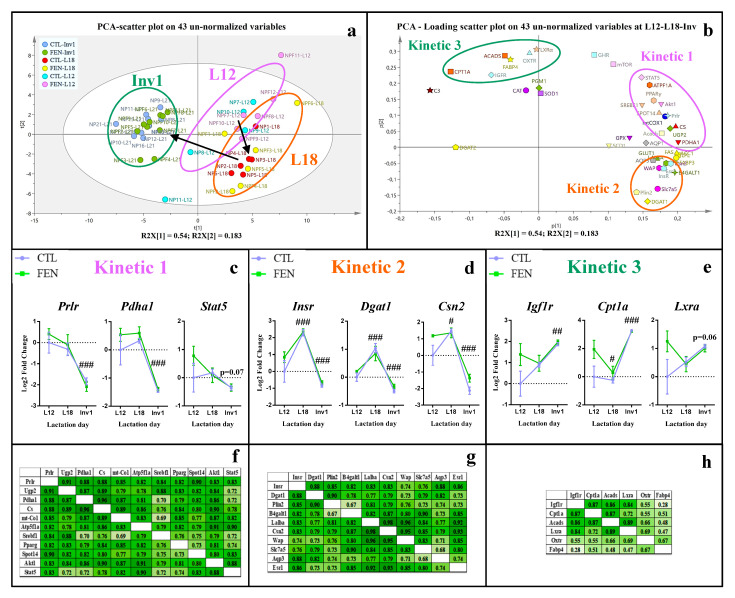
Temporal pattern of mammary gland gene expression in CTL and FEN groups through lactation and until involution. (**a**) Score plot on the first two dimensions of the PCA, accounting for 71% of the total variance for 43 un-normalized data (genes), (**b**) PCA loading plot presented clusters of variables highly associated to L12- and L18-groups (kinetic 1 and 2 clusters) or to Inv1 group (kinetic 3 cluster). Temporal pattern expression of representative genes of kinetic 1 (**c**), kinetic 2 (**d**) and kinetic 3 (**e**) clusters. Values were mean ± SEM and were analyzed with 2-way ANOVA followed by Tukey’s post-hoc tests. #: *p* < 0.05, ##: *p* < 0.01, ###: *p* < 0.001 for comparison with previous period. Correlations between gene expression in cluster 1 (**f**), 2 (**g**) and 3 (**h**) were analyzed with Pearson tests. The shade of green corresponds to the strength of the correlation between the variables with a light green for a weak correlation and a dark green for a strong correlation. CTL: control diet group and FEN: fenugreek-supplemented diet group throughout the lactation period. L12 and L18: post-natal day of lactation 12 and 18, InV1: first day of involution

Following the unsupervised Principal Component Analysis (PCA) on the un-normalized complete set of 43 genes, the PCA score plot, on components 1 and 2, revealed natural longitudinal classification with differences corresponding to the three lactation time points and a clear-cut separation between involution (Inv1) and lactation period (L12 and L18) (Figure 2a). The PCA loading scatter plot (Figure 2b) reflected relationships between variables in this model, deciphering three clusters that we labelled “kinetics 1, 2 and 3”. In agreement with this longitudinal pattern, the univariate statistical analysis showed that expression of all genes evolves significantly between the middle and end of lactation, except for *Ghr* and *Mtor* (Table 4 and Table 5).

Most of the genes studied exhibited a significant decrease in their expression between L18 and Inv1 ranging from 1.46-fold (*p* = 0.010) for *Atp5f1a* to 36.91-fold (*p* < 0.001) for *Fasn* and belonged to “***kinetic 1***” and “***kinetic 2***” patterns.

In the “kinetic 1” pattern, genes similarly expressed or only slightly modulated between L12 and L18, suggesting they had reached peak expression in this interval (Figure 2c). This pattern was represented mostly by genes coding for regulation of milk nutrient synthesis, energy metabolism, and galactose uptakes (*Stat5*, *Akt1*, *Spot14*, *Pparg*, *Prlr*, *Pdha1*, *Acacb*, *Mtco1*, *Cs*, *Ugp2* and *Atp5f1a*). All these genes were highly correlated with each other, as validated by Pearson’s test (Figure 2f and Supplementary Figure S1) and mainly with *Prlr* (r ≥ 0.8), considered as the barycenter of the “***kinetic*** 1” cluster in the PCA-loading scatter plot (Figure 2b). If not in the “kinetic 1” ellipse in the PCA (Figure 2b) genes related to de novo fatty acid synthesis and glucose uptake such as *Lpl*, *Fasn*, *Acaca*, *Fabp3* and *Glut1* were also strongly correlated with Prlr (r ≥ 0.7) (Supplementary Figure S2).

In “***kinetic 2***”, overall gene expression rose from 1.70-fold (*p* = 0.042) for *Csn2* to 2.62-fold for *Aqp3* (*p* = 0.001) between L12 and L18, suggesting that peak expression of these genes occurred closer to L18 than L12 (Figure 2d). This cluster mainly included genes related to milk constituent secretion (*Slc7a5*, *Wap*, *Csn2*, *B4galt1*, *Lalba*, *Plin 2*, *Aqp3*, *InsR*, *Esr* and *Dgat1*) as illustrated in Figure 2b,d,g, with *Insr*, closest to the barycenter of the “***kinetic 2***” cluster (Figure 2b), and strongly correlated with all these genes (Figure 2g). Otherwise, *Esr1* was probably linked to protein expression as its expression was very strongly correlated with all main milk protein genes (r ≥ 0.85).

In “***kinetic 3***”, contrary to other clusters, genes were significantly overexpressed at Inv1 compared to L18 (Figure 2e), with a rise ranging from 1.37-fold (*p* < 0.001) for *Pgm1* to 9.44-fold (*p* < 0.001) for *Cpt1a*. Several of these genes involved in fatty acid oxidation pathway (*Cpt1a*, *Acads*, *Lxra* and *Igf1r*) presented high correlation between each other (Figure 2h).

In the pituitary gland, the peak of expression was around L12 for Drd2, between L12 and L18 for Ghrhr and Prl, around L18 for Vipr2 and Trhr and around Inv1 for Esr1 (Table 6).

**Figure 3:**
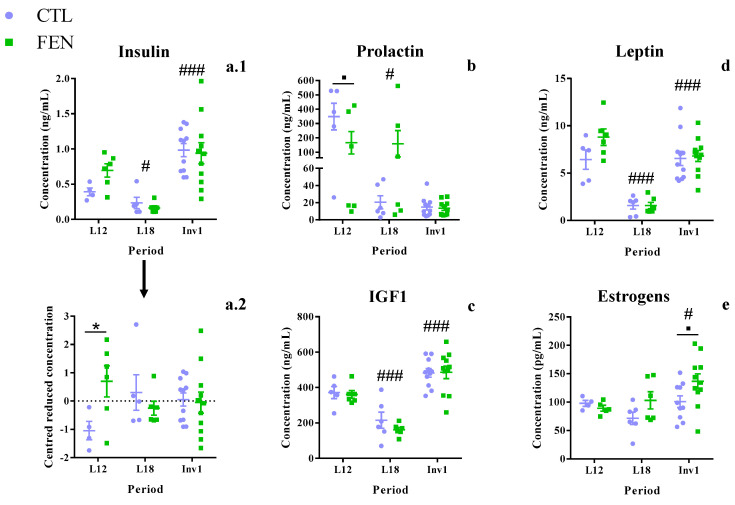
Effect of fenugreek on the concentration of insulin (**a.1**,**a.2**), prolactin (**b**), IGF-1 (**c**), leptin (**d**) and estrogens (**e**) in maternal plasma along the lactation period. Figure 3a.2 represents insulin concentration centered and reduced by period. Values are mean ± SEM with *n* = 4–6 at L12 and L18 and *n* = 11 at Inv1. Data were analyzed with 2-way ANOVA followed by Sidak’s post-hoc test for diet effect and Tukey’s post-hoc test for period effect: *p* < 0.1 and *: *p* < 0.05 for diet comparison; #: *p* < 0.05, and ###: *p* < 0.001 for comparison between a given period and the immediately preceding period. CTL: control diet group and FEN: fenugreek-supplemented diet group throughout the lactation period. L12 and L18: post-natal day of lactation 12 and 18, InV1: first day of involution.

Insulin, IGF-1 and leptin maternal plasma concentrations exhibited the same time course (Figure 3) with a significant decline between L12 and L18 (−65.9%, *p* = 0.030; −48.5%, *p* < 0.001; −79.7%, *p* < 0.001, respectively) followed by a sharp rise between L18 and Inv1 (+392.5%, *p* < 0.001; +156.9%, *p* < 0.001; +325.0%, *p* < 0.001, respectively), regardless of maternal diet. Plasma prolactin concentration decreased by 64.0% (*p* = 0.012) between L12 and L18. Finally, plasma estrogen concentration rose 37.1% (*p* = 0.029) between L18 and Inv1.

### 3.3. Fenugreek Supplementation Impacts Mammary Gland Metabolic Pathways Differently during Lactation and at Involution

**Figure 4:**
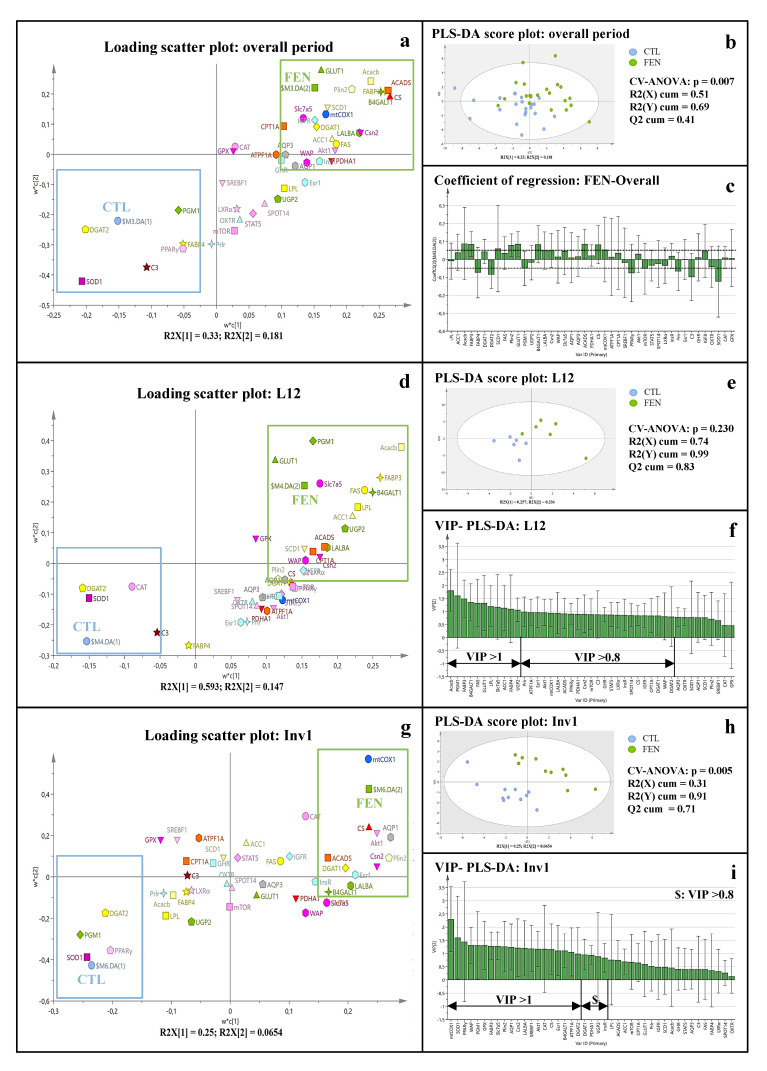
Fenugreek supplementation impacts mammary gland gene expression in CTL and FEN groups, regardless of lactation stage. PLS-DA score plot (on the first two components) built on all normalized variables (that have been mean-centered and scaled by lactation time point), revealed an effect of fenugreek supplementation on mammary gland gene expression during the entire period of lactation (**b**), at L12 (**e**) and Invl (**h**). PLS-DA loading plot presented clusters of variables highly associated to FEN and CTL groups’ overall lactation (**a**), at L12 (**d**) and Invl (**g**) with high regression coefficient (i.e., high prediction vector) for FEN class overall lactation (**c**) or high VIP scores on the two first PLS-DA components, at L12 (**f**) and Inv1 (**i**). CTL: control diet group and FEN: fenugreek-supplemented diet group throughout the lactation period. L12 and L18: post-natal day of lactation 12 and 18, InV1: first day of involution.

A supervised Partial Least Squares-Discriminate Analysis (PLS-DA), built on the same original dataset as PCA, showed that the stage of lactation had more impact than diet on the first two components (Supplementary Figure S1). Thus, a mean-centering and standardization by lactation stage of all variables was performed to assess the effect of diet per se. The resulting PLS-DA score plot revealed a clear-cut separation of diet groups (Figure 4b) overall. Most of the genes studied contributed to diet separation with a VIP over 0.8. *Acacb*, *Fabp3*, *Plin2*, *Glut1*, *B4galt1*, *Acads*, *Cs*, *Scd1*, *Lalba*, *Csn2*, *Slc7a5*, *Mtco1* and *Igf1r* were the genes the most associated to the FEN diet while *Sod1*, *Fabp4*, *Dgat2*, *Pparg*, *Prlr*, *C3* and *Pgm1* were the most associated to the CTL diet (Figure 4a,c).

Fenugreek modulated mammary gland gene expression mostly at L12 and Inv1. By focusing on each lactation time point with the PLS-DA, the predictive ability of the L12- (Figure 4e) and Inv1 (Figure 4h)-PLS-DA models was more important (R2(Y) cum and Q2 cum values above 0.9 and 0.7, respectively) than that of L18-PLS-DA model (Q2 cum = 0.39). At L12, the genes contributing the most to the separation of diets were *Acacb*, *Fabp3*, *Fasn*, *Glut1*, *Pgm1*, *B4galt1*, *Slc7a5*, associated with the FEN diet, but also *Fabp4*, *Dgat2* and *C3* associated to the CTL diet (VIP > 0.8, Figure 4d,f). Moreover, most of the genes overexpressed in the FEN diet at L12 were related to the synthesis of milk constituents, energy metabolism and the regulation of milk secretion (Table 2, Table 3, Table 4 and Table 5). At Inv1, some genes contribute strongly to the separation of both diets such as *Mtco1*, *Cs*, *Aqp1*, *Akt1*, *Cat*, *Plin2* and *Csn2* associated with the FEN diet, but also *Lpl*, *Dgat2*, *Pgm1*, *Ugp2*, *Pparg* and *Sod1* associated with the CTL diet (Figure 4g,i). Genes associated with the FEN diet were mostly related to milk secretion and energy metabolism.

At L18, diets were poorly separated from each other by PLS-DA and no difference was observed in mammary gene expression between CTL and FEN diets.

#### 3.3.1. Fenugreek Supplementation Increases the Expression of Mammary Genes Involved in Lipid, Lactose and Protein Synthesis

The effect of fenugreek was studied individually on genes involved in the synthesis of milk constituents (Table 2). The FEN diet significantly increased the overall expression of genes related to milk lipid synthesis (*Lpl*, *Fas*, *Acaca*, *Fabp3*), lactose synthesis (*Glut1*, *Ugp2*, *B4galt1*, *Lalba*) and protein synthesis (*Lalba* and *Csn2*) (Table 2). Moreover, *Acacb*, *Fabp3*, *Plin2*, *Glut1*, *B4galt1*, *Scd1*, *Lalba*, *Csn2* and *Slc7a5* were strongly associated with the FEN diet in the overall PLS-DA (VIP > 1, Figure 4a,c) and highly predictive of FEN group (regression coefficient ≥ 0.5).

#### 3.3.2. Fenugreek Supplementation Increases Expression of Genes Involved in Fuel Metabolism, Particularly Fatty Acid β-Oxidation

The overall expression of *Cpt1a*, *Acads* and *Cs* was significantly increased with the FEN diet compared to the CTL diet, with a particular overexpression of lipid β-oxidation-related genes, *Cpt1a* and *Acads*, at L12: 3.84-fold and 2.77-fold (*p* < 0.001), respectively (Table 3). Moreover, *Acads*, *Cs* and *Mtco1* were among the most powerful discriminators associated with the FEN diet in the overall PLS-DA (VIP > 0.8, Figure 4a,c).

#### 3.3.3. Fenugreek Increases the Expression of Regulatory Factors Involved in Protein and Lipid Metabolism

The FEN diet had no significant overall effect on the gene expression of factors regulating milk protein and lipid synthesis (Table 4) but induced an early transitory significant overexpression of *Lxra* and *Mtor* at L12 (2.38-fold, *p* = 0.01 and 1.84-fold, *p* = 0.032, respectively) contributing, with *Pparg*, to discrimination of the FEN diet group in the PLS-DA model at L12 (VIP > 0.8, Figure 4d,f) Additionally, *Pparg* and *Akt1* were significantly upregulated at L12 compared to L18 (1.85-fold, *p* = 0.046 and 1.62-fold, *p* = 0.035, respectively) in the FEN diet, whereas similar expression was observed for the CTL diet (*p* ≥ 0.98).

#### 3.3.4. Fenugreek Upregulates the Expression of Insulin, GH and IGF-1 Receptors

The effect of fenugreek was studied on receptors of the main lactogenic hormones (Table 5). *Igf1r* expression was the most enhanced by the FEN diet throughout lactation (*p* = 0.033; Figure 4a,c), specifically at L12 (2.60-fold; *p* = 0.012 and VIP > 0.8, Figure 4d), with a similar trend observed for *Insr* and *Ghr* expressions (VIP > 0.8, Figure 4d). Interestingly, normalized gene expression of *Igf1r* was strongly correlated with those of *Ghr* and *Insr* overall lactation (r = 0.76 and 0.71 respectively, Supplementary Figure S3).

### 3.4. Fenugreek Stimulates Oxytocin Expression at the Pituitary Level

The FEN diet increased the overall expression of *Oxt*, *C3* and *Trhr*, mostly at L18 for *Oxt* and C3 (*p* = 0.029 and *p* = 0.004, respectively), and tended to increase the expression of *Vipr2*. In addition, *Drd2* was overexpressed in the FEN group at Inv1 (*p* = 0.050), whereas it was on average under-expressed in this group during lactation (interaction: *p* = 0.040).

### 3.5. Fenugreek Supplementation Increases Plasma Insulin Concentration

The FEN diet had no significant overall impact on the concentration of these hormones (Figure 4). However, plasma estrogen concentration tended to be higher in the FEN compared to CTL group (*p* = 0.082), particularly at Inv1 (+35.5%, *p* = 0.056). Moreover, variation of insulin concentration was not homogenous between lactation and involution (*p* = 0.035 for the Brown–Forsythe test), impairing our ability to detect any effect of supplementation during lactation. By centering and reducing insulin concentration by time period, a rise in insulin concentration was observed in the FEN group at L12 (+77.1%, *p* = 0.043), whereas no difference was observed at L18 and Inv1 (interaction: *p* = 0.040). Finally, a significant interaction was observed for prolactin concentration explained by the precipitous drop between L12 and L18 in the CTL group (94.1%, *p* < 0.001), whereas it remained steady in the FEN group (*p* = 0.995). However, the large prolactin concentration variations observed during lactation (mean CV of 102.1% and interval of (2.87; 528.2) ng/mL) makes it difficult to draw any conclusion from these values.

## 4. Discussion

The findings of the current study confirm that fenugreek increases milk production in a model of rat nursing a large litter. They further demonstrate that the impact of dietary fenugreek supplementation on milk production is associated with a stimulation of milk macronutrient synthesis and mammary fuel metabolism. These effects were mainly observed at L12 and Inv1 but not at L18, suggesting that fenugreek promotes an earlier peak of lactation and maintains milk production longer, whereas it does not seem to have further beneficial effect on milk macronutrient synthesis once peak lactation is reached. Our findings finally suggest that the effect of fenugreek on milk production at mid-lactation may be mediated by a stimulation of insulin secretion and a modulation of the insulin/GH/IGF-1 axis, while its action on maintaining lactation until the first day of mammary involution could be due to its estrogenic effect.

### 4.1. The Time Course of the Gene Expression in Mammary Gland and Lactogenic Hormones Suggests Peak Lactation Is Closer to L18

A noticeable result is the importance of lactation stage on mammary gland gene expression, as illustrated by the clear-cut separation of lactation time points in both PCA and PLS-DA models (Figure 2a and Supplementary Figure S1). The first day of involution (Inv1) presented clearly different gene expression pattern from those at mid- and late-lactation.

Most genes, particularly those following ***kinetics 1*** and ***2*** (Figure 2c,d), were downregulated during involution, implying they were overexpressed at mid- and late-lactation, highlighting their central role for milk production and regulation [32]. Genes following ***kinetic 1*** were related to the first steps of milk lipid and lactose synthesis [19], such as glucose and fatty acid uptake (*Glut1*, *Lpl*), fatty acid de novo synthesis (*Fasn*, *Acaca*) and galactose synthesis (*Ugp2*), to mammary energetic metabolism (*Cs*, *Mtco1*) and to milk synthesis regulatory factors (*Stat5*, *Akt1*, *Pparg*). These genes reached their peak expression between mid- and late-lactation and were probably under primary regulation of *Prlr* whose expression was highly correlated with all (r > 0.8). Genes following ***kinetic 2*** were associated with the final steps of milk constituent secretion [19], such as triglyceride synthesis (*Scd1*, *Dgat1*), lipid droplet formation (*Plin2*), lactose synthesis (*B4galt1*, *Lalba*), main milk protein synthesis (*Csn2*, *Wap*) and water inflow (*Aqp1*, *Aqp3*). They seemed to be mostly controlled by *Insr* (*r* ≥ 0.75 with all) but also *Esr1,* mainly for milk protein genes (r ≥ 0.85). To the contrary, genes following ***kinetic 3*** (Figure 2e) were upregulated at Inv1, suggesting that their expression was repressed during lactation. These genes are related to metabolic pathways repressed during lactation such as mitochondrial β-oxidation (*Acads*, *Cpt1a*) [32,33,42] or have a secondary role on regulation (*Igf1r*, *Lxra*) [33,43,44] or on lipid synthesis (*Fabp4*, *Dgat2*) [33,45] compared to other regulatory factors (*Prlr*, *Srebf1*) or other family members (*Fabp3*, *Dgat1*). Moreover, in the present study, the drop in the expression of de novo fatty acid synthesis genes at involution is correlated (Supplementary Figure S2) with the concomitant increase in the expression of β-oxidation genes likely related to energy requirements for mammary tissue remodeling. Finally, *Oxtr* was overexpressed in involuting mammary gland probably because myoepithelial cells were preserved longer than lactocytes and contracted to facilitate removal of degenerating epithelial cells [46].

Interestingly, genes related to milk constituent secretion followed the ***kinetic 2*** pattern, and as such, were overexpressed at L18 compared to L12. These genes are probably the more strongly correlated with actual milk secretion, suggesting that, among the three periods studied, milk production was likely at its maximum at L18. This is consistent with (i) the time course of plasma insulin, leptin and IGF-1, which all declined to their minimal concentration at L18. Indeed, the plasma concentration of these hormones is known to decline during lactation partly due to increased hormone extraction by the mammary gland [47,48,49] and they are negatively correlated with milk production [50]. (ii) The highest milk consumption by rat pups reported to occur at L15-L16 as they only start to consume solid food significantly at L17 and drinking water at L19 [51].

### 4.2. Fenugreek Supplementation Stimulates Milk Macronutrient Synthesis

Dietary fenugreek supplementation increased the overall expression of genes involved in fatty acid uptake (*Lpl*), de novo synthesis (*Fasn*, *Acaca*, *Acacb*), transport (*Fabp3*) and, to a lesser extent, lipid secretion (*Plin2*). Fenugreek also stimulated the expression of genes involved in glucose uptake (*Glut1*), galactose (*Pgm1*, *Ugp2*) and lactose (*B4galt1*, *Lalba*) synthesis. Finally, fenugreek increased the expression of amino acid transporters (*Slc7a5*) and main milk proteins (*Csn2*, *Lalba*). These results suggest an increase in the synthesis of the 3 macronutrients of milk namely lipid, lactose and protein in the mammary gland [19] and are consistent with the stimulating effect of fenugreek on milk flow by 16% reported in our earlier study [15] and 15.3% in this study. More specifically, genes involved at every step of protein and lactose synthesis were significantly overexpressed, whereas only genes involved in the fatty acid synthesis were dramatically overexpressed for lipid synthesis. The expression of genes involved in triglyceride synthesis and lipid secretion was not or only slightly affected. This relative lack of conversion of fatty acids to triglycerides may account for the decrease in milk lipid concentration found in some fenugreek supplementation studies [52,53,54]. Otherwise, the stimulation by fenugreek of genes involved in mitochondrial energy metabolism (*Cs*, *Atp5f1a*, *Mtco1*) and particularly those involved in β-oxidation (*Cpt1a*, *Acads*) suggests that increased ATP synthesis took place in the mammary gland to meet the increased energy requirements associated with increased milk production [22]. Nevertheless, contrary to Liu et al. [55], we were unable to observe a significant increase in water inflow in response to galactagogue even though mean *Aqp1* and *Aqp3* expressions were increased at L12 and L21.

### 4.3. Fenugreek Supplementation Advances Peak Lactation and Maintains Milk Production

The lack of separation according to diet on the PLS-DA plot, and the lack of any difference in the expression of genes related to milk constituent synthesis and fuel metabolism in FEN and CTL groups at L18, suggests that the milk synthesis peak observed at late-lactation mainly in control dams was not impacted by fenugreek supplementation. However, the time course of some genes from L12 to L18 differed between diets: either the gene expression increased in the CTL diet and did not change in the FEN diet (*Lpl*, *B4galt1*, *Slc7a5*, *Csn2* and *Aqp1*), or the expression did not change in the CTL diet and decreased in the FEN diet (*Acaca*, *Fabp3*, *Ugp2*, *Cpt1a*, *Acads*). In both cases, gene expression was higher earlier in the FEN group. This is consistent with the clear separation between both diets observed in the PLS-DA model built on gene expression at L12 (Figure 4d) and the fact that greater overexpression was observed at L12 for most of the genes in the FEN group. Finally, the PLS-DA model built on gene expression at Inv1 also properly separated both diets and the expression of the genes *Lalba*, *Csn2*, *Plin2*, *Slc7a5*, *Aqp1* and *Esr1* was maintained to a slightly greater level in the FEN diet. Together these results suggest that fenugreek supplementation advanced the peak of milk production, observed near L18 for control, around L12 and maintain it to a level similar to control at L18. Thus, fenugreek supplementation may enable the dam to maintain a milk production slightly higher than control during involution.

### 4.4. Fenugreek Stimulates Milk Production Mainly by Modulating Insulin and Oxytocin Secretion

The main effect of fenugreek on the expression of genes related to milk synthesis was observed at L12. During the same period, fenugreek supplementation increased dams’ plasma insulin by 77.1% and the expression of *Insr* by 1.79-fold, suggesting an effect of fenugreek on insulin secretion and its action in mammary gland. Receptors to GH and IGF-1 (GHR and IGF1R) were also remarkably overexpressed in the mammary gland at L12 (Table 5). These 3 hormones are closely linked by the insulin/GH/IGF-1 axis [56]. Briefly, GH is secreted by pituitary gland and stimulates IGF-1 secretion in the liver [56,57], which shares strong homology with insulin. Both hormones can bind to IGF1R and insulin receptors (INSR) and to hybrid formed by both receptors with more or less affinity. These receptors act through similar pathway mainly by activating the AKT/mTOR pathway [56]. In addition, insulin stimulates expression of GH receptor in peripheral tissues [58] but also modulates the expression of IGF1R and INSR [59,60].

**Figure 5:**
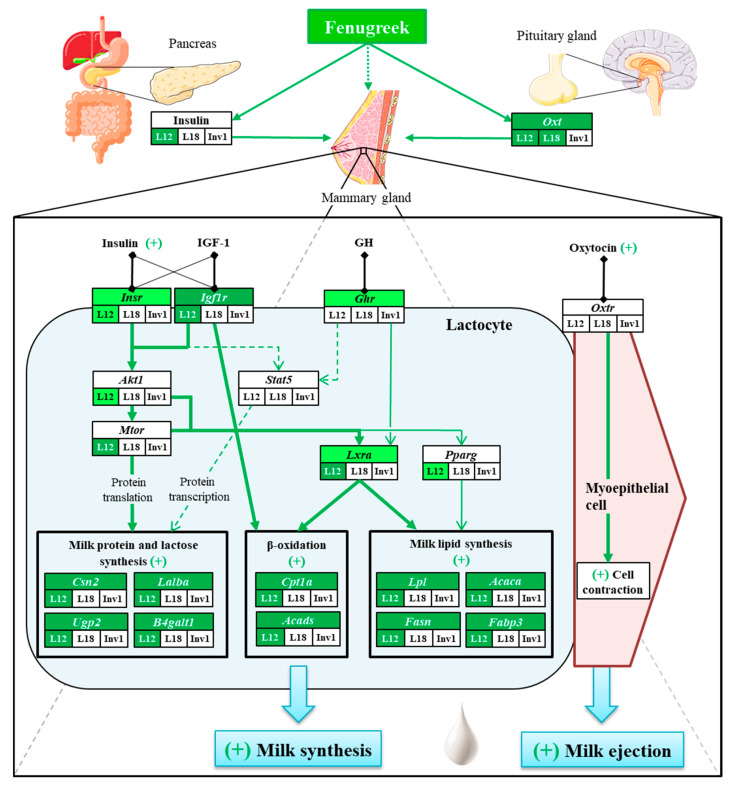
Suggested mechanisms of action of fenugreek on milk synthesis and secretion. Green arrows denote positive activation, bold and thin arrows represent main and secondary fenugreek action pathways, respectively. Dotted lines represent hypothetical pathway of action. Dark and light green boxes represent significant or trend increase respectively of gene expression or plasma concentration overall for upper box and at each lactation period for lower boxes. White boxes depict the lack of modulation by fenugreek.

We therefore hypothesize that fenugreek acts mainly by increasing insulin secretion by maternal pancreas β-cells. This is consistent with the hypoglycemic effect of fenugreek and its capacity to stimulate insulin secretion through the potential action of trigonelline and 4-hydroxyisoleucin [23,24]. Together with a potential direct action of fenugreek compounds [24], the rise in plasma insulin under lactation-specific conditions, i.e., hypo-insulinemia and insulin-resistance in peripheral tissues except mammary gland, could explain the *Igf1r*, *Insr* and *Ghr* overexpression in the mammary gland [58,60,61]. The increase of IGF-1 and insulin binding to their receptor and to their hybrid receptor leads to an activation of the AKT/mTOR pathway, as suggested by overexpression of *Mtor* at L12 (by 1.84-fold) and of *Akt1* to a lesser extent (Table 4) and results in activation of lactose and protein synthesis [19]. GHR could also act secondarily on protein and lactose synthesis through the JAK2/STAT5 pathway [62]. Concerning lipid metabolism, *Lxra* and *Pparg* are the most overexpressed regulatory factors with fenugreek supplementation (Table 4). Both are stimulated by insulin [63,64,65] and likely IGF-1, and *Lxra* expression is also stimulated by GH [64]. *Lxra* has been shown to stimulate de novo fatty acid in the mammary gland [66] independently of SREBF1 but not fatty acid desaturation and triglyceride synthesis [44], which is consistent with fenugreek effect found only for fatty acid uptake and de novo synthesis. *Lxra* was also related to activation of lipolysis in adipocyte [63,64] and may be responsible for the maintenance of β-oxidation-related genes at L12, as suggested by the strong correlation with *Acads* and *Cpt1a* (r > 0.7, Figure 2e). *Pparg* had a secondary milk lipid regulatory role in rodent compared to ruminant [19] but promote de novo fatty acid synthesis, triglyceride synthesis and lipid secretion [19,45,65]. Finally, *Igf1* is known to be an important activator of fatty acid β-oxidation [56] that could counteract insulin inhibitory effect [20,42] on this pathway and notably at involution. It may act independently on the maintenance of *Acads* and *Cpt1a* expression, as suggested by the very strong correlation between *Igf1r*, *Acads* and *Cpt1a* expressions (r ≥ 0.84, Figure 2e). Suggested mechanisms associated to the galactologue effect of fenugreek are summarized in Figure 5.

Another mechanism by which fenugreek could increase milk flow may be its capacity to favor milk ejection by stimulating oxytocin secretion [22]. Indeed, fenugreek is a well-known oxytocic agent used to promote uterine contractions at delivery [23,25] and oxytocin expression was overexpressed in the pituitary gland during lactation in the fenugreek-supplemented diet, particularly at L18 (1.88-fold). Even if *Oxtr* expression was not significantly increased in mammary gland, fenugreek might maintain greater milk flow at L18 by promoting milk ejection (Figure 5).

Nevertheless, we could not observe increase in mother prolactin, as suggested in previous studies [26,53], because of strong variability in plasmatic prolactin concentration. These variations were probably due to the too-short dam/pup separation time before sacrifice (60 min) [67], which made prolactin concentration more likely representative to the time since last suckling.

### 4.5. Fenugreek Stimulates Estrogenic Activity at the End of Lactation and Increased Energy Availability for Milk Production

At L18 and Inv1, fenugreek-supplemented dams tended to have higher plasma estrogens, and in the pituitary, had higher expression of C3, which is known to be a strong estrogenic biomarker in the uterus [68]. Moreover, *Esr1* tended to be upregulated in the mammary gland at L21 (Figure 3g) and was strongly correlated with genes overexpressed at this period (*r* ≥ 0.74 with *Csn2, Lalba, Aqp1, Plin2* and *Akt1*, Figure 2g and Supplementary Figure S1). As fenugreek is known to contain several estrogenic compounds [23,27], those might contribute to maintaining the expression of milk secretory genes at Inv1 partly through the mediation of *Akt1* [69].

Finally, fenugreek supplementation increased global food consumption of dams by 10%, in line with earlier studies [52,70,71], principally at mid-lactation (Supplementary Figure S4) that could partly account for fenugreek lactogenic effect. Indeed, lactation is a period of high energy requirement characterized by an important hyperphagia, in particular in small animals where food intake can be increased by 450% [72]. Rats have little lipid storage during lactation and energy requirement for milk production almost exclusively comes from increased food consumption [22,73]. Thus, by enhancing food consumption, fenugreek might enhance energy availability for milk production. This increase in food intake observed after fenugreek supplementation might be mediated (i) by a family of its bioactive compounds, steroid saponins, that stimulate eating motivation [74,75] and could act directly on the hypothalamus or through stimulation of prolactin, or (ii) by a greater peak of prolactin during suckling, as suggested by overexpression of receptors of prolactin secretion activators in the pituitary gland (thyrotropin releasing hormone and vasoactive intestinal peptide) [76]. Indeed, lactation hyperphagia is under the primary control of prolactin which decreases leptin secretion in adipocytes and, in the hypothalamus, stimulates orexigenic neuropeptide Y secretion and decreases its sensitivity to leptin [22]. Moreover, milk production is also known as an important determinant of food intake in rat [72,77]. Indeed, greater milk yield leads to higher dam’s energy expenditure and likely, enhanced suckling stimulus, as mammary gland was reported to be emptied at each feeding for a rodent litter-size model of 12 pups [78], resulting in greater food intake. This is in accordance with the peak dietary intake observed when fenugreek dams started their peak milk production.

Otherwise, we observed higher leptin and insulin level in plasma of fenugreek-supplemented dams likely caused by fenugreek stimulation of insulin secretion [79]. Yet, these hormones are known to exhibit important anorexigenic effect under normal physiological conditions [80]. However, during lactation, because of central resistance to their signals [22], insulin and leptin had only secondary roles in regulating lactation-associated hyperphagia compared to suckling stimulus and prolactin action [77,81]. Moreover, under normal conditions, orexigenic effect of fenugreek was observed despite an increase of plasma insulin [74]. Thus, effect of fenugreek on food intake was probably not related to the increase of both insulin and leptin plasma concentrations.

Whatever the cause, overall food increase in fenugreek-supplemented dams was of 10% and only of 5.3% (*p* = 0.041) related to dams’ body weight, so it cannot alone account for the 15% increase in milk production observed in our study.

## 5. Conclusions

The current study confirms the ability of dietary fenugreek supplementation to promote milk production, and sheds light into its action at the molecular level. Indeed, milk flow was increased by 15.3% and most of the genes related to milk lipid, lactose and protein synthesis as well as energy metabolism were overexpressed. Moreover, our findings suggest that fenugreek may act by extending the duration of peak lactation until the beginning of involution rather than intensifying it. According to our data, the main mechanism of action of fenugreek on milk production is likely through activation of the insulin/GH/IGF-1 axis, which leads to a greater mammary sensitivity to these hormones. Consequently, milk protein and lactose synthesis could be mainly activated by the AKT/mTOR pathway, while milk lipid synthesis and energetic metabolism could be stimulated by LXRα, PPARγ and probably IGF1R itself. Moreover, fenugreek would stimulate milk ejection by an increase of pituitary oxytocin secretion. However, data from gene expression must be confirmed by proteomic and histologic analysis.

## Figures and Tables

**Table 1:** Effect of fenugreek supplementation on dams’ lactation performances.

Groups	*N*	Dam Water Intake (g/day)	Dam Food Intake (g/day)	Pup Total Weight Gain (g)	Milk Flow (g/day)
CTL	17	53.93 ± 1.12	49.71 ± 0.79	36.19 ± 0.74	55.90 ± 2.36
FEN	16	55.22 ± 1.11	54.66 ± 1.00	38.75 ± 0.85	64.45 ± 2.35
*p*-value		0.422	**<0.001**	**0.029**	**0.016**

Values are mean ± SEM (standard error of the mean) and were analyzed with Student’s test. Data were obtained from pooled Xp1 and Xp2 experiments. CTL: control diet group and FEN: fenugreek-supplemented diet group throughout the lactation period. The values in bold correspond to significant *p*-values (<0.05).

**Table 2:** Effect of fenugreek supplementation on relative expression of milk constituent synthesis genes in mammary gland along lactation period.

		Log2 (Fold Change) CTL at L12 as Reference		Fold Change CTL as Reference	2-Way ANOVA		
Genes	Period						
		CTL	FEN		Inter	Period	Diet
Milk lipid synthesis							
	L12 (*n* = 6)	0.00 ± 0.27 **^a,1^**	0.97 ± 0.11 **^b,1^**	1.95			
*Lpl*	L18 (*n* = 6)	0.52 ± 0.13 **^1^**	0.90 ± 0.43 **^1^**	1.31	**0.034**	**<0.001**	**0.045**
	Inv1 (*n* = 11)	−2.63 ± 0.16 **^2^**	−2.83 ± 0.16 **^2^**	0.87			
	L12	0.00 ± 0.27 **^a,1^**	1.10 ± 0.13 **^b,1^**	2.14			
*Fasn*	L18	0.21 ± 0.17 **^1^**	0.25 ± 0.37 **^1^**	1.03	0.192	**<0.001**	**0.049**
	Inv1	−5.10 ± 0.23 **^2^**	−4.84 ± 0.29 **^2^**	1.19			
	L12	0.00 ± 0.32 **^a,1^**	1.08 ± 0.14 **^b,1^**	2.12			
*Acaca*	L18	−0.18 ± 0.14 **^1^**	0.16 ± 0.41 **^2^**	1.26	0.078	**<0.001**	**0.012**
	Inv1	−4.24 ± 0.14 **^2^**	−4.19 ± 0.18 **^3^**	1.04			
	L12	0.00 ± 0.16 **^1^**	0.31 ± 0.08 **^1^**	1.24			
*Acacb*	L18	0.52 ± 0.20 **^1^**	0.43 ± 0.31 **^1^**	0.94	0.480	**<0.001**	0.965
	Inv1	−2.12 ± 0.18 **^2^**	−2.37 ± 0.26 **^2^**	0.84			
	L12	0.00 ± 0.27 **^a,1^**	1.84 ± 0.23 **^b,1^**	3.59			
*Fabp3*	L18	0.51 ± 0.16 **^1^**	0.67 ± 0.38 **^2^**	1.12	**0.009**	**<0.001**	**<0.001**
	Inv1	−4.37 ± 0.16 **^2^**	−3.95 ± 0.26 **^3^**	1.34			
	L12	0.00 ± 0.24 **^1^**	−0.03 ± 0.17 **^1^**	0.98			
*Fabp4*	L18	−1.73 ± 0.29 **^2^**	−1.75 ± 0.48 **^2^**	0.99	0.959	**<0.001**	0.759
	Inv1	−0.30 ± 0.16 **^1^**	−0.44 ± 0.16 **^1^**	0.91			
	L12	0.00 ± 0.71	1.19 ± 0.16 **^1,2^**	2.28			
*Scd1*	L18	1.10 ± 0.18	2.10 ± 0.41 **^1^**	2.00	0.399	**0.013**	0.122
	Inv1	0.04 ± 0.52	−0.03 ± 0.56 **^2^**	0.95			
	L12	0.00 ± 0.15 **^1^**	0.21 ± 0.03 **^1^**	1.16			
*Dgat1*	L18	1.04 ± 0.17 **^2^**	0.85 ± 0.26 **^2^**	0.88	0.273	**<0.001**	0.564
	Inv1	−0.50 ± 0.06 **^3^**	−0.33 ± 0.08 **^3^**	1.12			
	L12	0.00 ± 0.36 **^1,2^**	−0.74 ± 0.20 **^1^**	0.60			
*Dgat2*	L18	−0.33 ± 0.10 **^1^**	−0.25 ± 0.42 **^1-2^**	1.05	0.068	**0.005**	0.322
	Inv1	0.65 ± 0.17 **^2^**	0.12 ± 0.23 **^2^**	0.69			
	L12	0.00 ± 0.43 **^1^**	0.50 ± 0.08 **^1^**	1.41			
*Plin2*	L18	1.51 ± 0.16 **^2^**	1.43 ± 0.26 **^2^**	0.95	0.267	**<0.001**	0.069
	Inv1	−0.37 ± 0.13 **^1^**	0.16 ± 0.16 **^1^**	1.44			
Milk lactose synthesis							
	L12	0.00 ± 0.37 **^1^**	0.49 ± 0.21 **^1^**	1.40			
*Glut1*	L18	−0.52 ± 0.14 **^1^**	−0.06 ± 0.29 **^1^**	1.38	0.310	**<0.001**	**0.031**
	Inv1	−2.08 ± 0.07 **^2^**	−2.04 ± 0.08 **^2^**	1.03			
	L12	0.00 ± 0.10 **^1^**	0.33 ± 0.06 **^1^**	1.26			
*Pgm1*	L18	−0.61 ± 0.11 **^2^**	−0.60 ± 0.18 **^2^**	1.01	**0.032**	**<0.001**	0.591
	Inv1	−0.05 ± 0.05 **^1^**	−0.26 ± 0.08 **^3^**	0.86			
	L12	0.00 ± 0.35 **^a,1^**	1.03 ± 0.13 **^b,1^**	2.05			
*Ugp2*	L18	−0.67 ± 0.24 **^1^**	−0.48 ± 0.44 **^2^**	1.14	**0.042**	**<0.001**	**0.039**
	Inv1	−2.49 ± 0.11 **^2^**	−2.56 ± 0.06 **^3^**	0.95			
	L12	0.00 ± 0.26 **^a,1^**	1.23 ± 0.13 **^b,1^**	2.35			
*B4galt1*	L18	0.74 ± 0.33 **^2^**	0.88 ± 0.27 **^1^**	1.11	**0.009**	**<0.001**	**0.001**
	Inv1	−3.22 ± 0.06 **^3^**	−3.06 ± 0.09 **^2^**	1.11			
	L12	0.00 ± 0.58 **^a,1^**	1.28 ± 0.12 **^b,1^**	2.42			
*Lalba*	L18	1.65 ± 0.15 **^2^**	1.64 ± 0.30 **^1^**	0.99	0.166	**<0.001**	**0.019**
	Inv1	−1.44 ± 0.28 **^3^**	−0.86 ± 0.25 **^2^**	1.49			
Milk protein synthesis							
	L12	0.00 ± 0.41 **^1^**	0.83 ± 0.11 **^1^**	1.78			
*Slc7a5*	L18	1.12 ± 0.25 **^2^**	0.74 ± 0.24 **^1^**	0.77	0.086	**<0.001**	0.164
	Inv1	−1.73 ± 0.16 **^3^**	−1.31 ± 0.26 **^2^**	1.34			
	L12	0.00 ± 0.60 **^1^**	1.18 ± 0.06 **^b,1^**	2.27			
*Csn2*	L18	1.48 ± 0.12 **^2^**	1.36 ± 0.30 **^1^**	0.92	0.104	**<0.001**	**0.013**
	Inv1	−2.13 ± 0.22 **^3^**	−1.37 ± 0.24 **^2^**	1.69			
	L12	0.00 ± 0.90 **^1^**	1.47 ± 0.11 **^1^**	2.77			
*Wap*	L18	1.58 ± 0.05 **^1^**	1.48 ± 0.28 **^1^**	0.93	0.329	**<0.001**	0.156
	Inv1	−2.29 ± 0.54 **^2^**	−1.91 ± 0.42 **^2^**	1.30			
Lactocyte water inflow							
	L12	0.00 ± 0.45 **^1^**	0.58 ± 0.11	1.50			
*Aqp1*	L18	1.24 ± 0.10 **^2^**	0.57 ± 0.26	0.63	**0.006**	**<0.001**	0.458
	Inv1	−0.39 ± 0.10 **^1^**	0.05 ± 0.14	1.36			
	L12	0.00 ± 0.38 **^1^**	0.63 ± 0.48 **^1^**	1.55			
*Aqp3*	L18	1.64 ± 0.35 **^2^**	1.82 ± 0.28 **^1^**	1.13	0.751	**<0.001**	0.240
	Inv1	−0.91 ± 0.30 **^1^**	−0.75 ± 0.17 **^2^**	1.12			

Log2 (fold change) corresponded to −ΔΔCq values calculated with mean of 3 housekeeping genes (*Actb*, *Uxt*, *Rps9*) and with CTL at L12 as a reference group. Fold change correspond to 2^−ΔΔCq^ values and were calculated each day for FEN diet with CTL of the day as a reference. Log2 (fold change) values were mean ± SEM, *n* = 5–6 at L12 and L18 and 10–11 at Inv1. Log2 (fold change) values were analyzed with 2-way ANOVA (*p*-values of diet, period and interaction effects reported in the last columns) followed by Sidak’s *post*-*hoc* test for diet effect and Tukey’s *post*-*hoc* test for period effect. Different letters and numbers represent significant difference (*p* < 0.05) between diets for each period and between periods for each diet, respectively. Green boxes represent significant overexpression in FEN group compared to CTL group at a given day. CTL: control diet group and FEN: fenugreek-supplemented diet group throughout the lactation period. L12 and L18: post-natal day of lactation 12 and 18, InV1: first day of involution. The values in bold correspond to significant *p*-values (<0.05).

**Table 3:** Effect of fenugreek supplementation on relative expression of genes involved in energy metabolism and antioxidant activity in mammary gland along lactation period.

		Log2 (Fold Change) CTL at L12 as Reference		Fold Change) CTL as Reference	2-Way ANOVA		
Genes	Period						
		CTL	FEN		Inter	Period	Diet
Lipid β-oxidation							
	L12 (*n* = 6)	0.00 ± 0.75 **^a,1^**	1.94 ± 0.64 **^b,1^**	3.84			
*Cpt1a*	L18 (*n* = 6)	−0.26 ± 0.53 **^1^**	0.29 ± 0.46 **^2^**	1.46	**0.021**	**<0.001**	**0.007**
	Inv1 (*n* = 11)	3.28 ± 0.05 **^2^**	3.22 ± 0.08 **^3^**	0.96			
	L12	0.00 ± 0.52 **^a,1^**	1.47 ± 0.40 **^b,1^**	2.77			
*Acads*	L18	0.19 ± 0.08 **^1^**	0.39 ± 0.13 **^2^**	1.15	**0.007**	**<0.001**	**0.002**
	Inv1	1.28 ± 0.06 **^2^**	1.41 ± 0.07 **^1^**	1.09			
Krebs cycle initiation							
	L12	0.00 ± 0.53 **^1^**	0.53 ± 0.23 **^1^**	1.45			
*Pdha1*	L18	0.32 ± 0.08 **^1^**	0.59 ± 0.22 **^1^**	1.21	0.465	**<0.001**	0.071
	Inv1	−1.46 ± 0.05 **^2^**	−1.39 ± 0.06 **^2^**	1.05			
	L12	0.00 ± 0.40 **^1^**	0.70 ± 0.25 **^1^**	1.62			
*Cs*	L18	0.10 ± 0.07 **^1^**	0.32 ± 0.20 **^1^**	1.16	0.246	**<0.001**	**0.013**
	Inv1	−1.20 ± 0.04 **^2^**	−1.03 ± 0.07 **^2^**	1.12			
Oxidative phosphorylation							
	L12	0.00 ± 0.53 **^1^**	0.74 ± 0.25 **^1^**	1.68			
*Mtco1*	L18	0.52 ± 0.17 **^1^**	0.35 ± 0.18 **^1^**	0.89	0.148	**<0.001**	0.099
	Inv1	−0.90 ± 0.07 **^2^**	−0.61 ± 0.12 **^2^**	1.22			
	L12	0.00 ± 0.50	0.55 ± 0.23 **^1^**	1.47			
*Atp5f1a*	L18	−0.24 ± 0.15	0.07 ± 0.18 **^1^**	1.24	0.251	**<0.001**	0.073
	Inv1	−0.61 ± 0.03	−0.64 ± 0.07 **^2^**	0.98			
Antioxidant enzymes							
	L12	0.00 ± 0.12 **^1^**	−0.24 ± 0.08	0.85			
*Sod1*	L18	−0.55 ± 0.14 **^2^**	−0.48 ± 0.18	1.05	0.285	**0.012**	0.142
	Inv1	−0.22 ± 0.08 **^1,2^**	−0.48 ± 0.08	0.83			
	L12	0.00 ± 0.18	−0.18 ± 0.10	0.88			
*Cat*	L18	−0.85 ± 0.13	−0.85 ± 0.21	1.00	0.458	**0.021**	0.726
	Inv1	−0.51 ± 0.28	−0.11 ± 0.23	1.32			
	L12	0.00 ± 0.18	0.20 ± 0.13 **^1^**	1.14			
*Gpx1*	L18	0.14 ± 0.13	0.38 ± 0.23 **^1^**	1.18	0.310	**<0.001**	0.680
	Inv1	−0.47 ± 0.17	−0.71 ± 0.17 **^2^**	0.85			

Log2 (fold change) corresponded to −ΔΔCq values calculated with mean of 3 housekeeping genes (*Actb*, *Uxt*, *Rps9*) and with CTL at L12 as a reference group. Fold change correspond to 2^−ΔΔCq^ values and are calculated each day for FEN diet with CTL of the day as a reference. Log2 (fold change) values are mean ± SEM, *n* = 5–6 at L12 and L18 and 10–11 at Inv1. Log2 (fold change) values were analyzed with 2-way ANOVA (*p*-values of diet, period and interaction effects reported in the last columns) followed by Sidak’s *post*-*hoc* test for diet effect and Tukey’s *post*-*hoc* test for period effect. Different letters and numbers represent significant difference (*p* < 0.05) between diets for each period and between periods for each diet, respectively. Green boxes represent significant overexpression in FEN group compared to CTL group at a given day. CTL: control diet group and FEN: fenugreek-supplemented diet group throughout the lactation period. L12 and L18: post-natal day of lactation 12 and 18, InV1: first day of involution. The values in bold correspond to significant *p*-values (<0.05).

**Table 4:** Effect of fenugreek on relative expression of genes related to milk synthesis regulatory factors in the mammary gland along lactation period.

		Log2 (Fold Change) CTL at L12 as Reference		Fold Change CTL as Reference	2-Way ANOVA		
Genes	Period						
		CTL	FEN		Inter	Period	Diet
Lipid metabolism regulatory factors							
	L12 (*n* = 6)	0.00 ± 0.37 **^1^**	0.40 ± 0.52 **^1^**	1.32			
*Srebf1*	L18 (*n* = 6)	−2.11 ± 0.29 **^2^**	−1.76 ± 0.62 **^2^**	1.27	0.605	**<0.001**	0.543
	Inv1 (*n* = 11)	−3.03 ± 0.18 **^2^**	−3.24 ± 0.21 **^3^**	0.87			
	L12	0.00 ± 0.46 **^1^**	0.77 ± 0.24 **^1^**	1.71			
*Pparg*	L18	−0.07 ± 0.28 **^1^**	−0.12 ± 0.39 **^2^**	0.97	0.097	**<0.001**	0.432
	Inv1	−0.93 ±0.10 **^2^**	−1.19 ± 0.10 **^3^**	0.84			
	L12	0.00 ± 0.49 **^1^**	0.55 ± 0.38 **^1^**	1.46			
*Spot14*	L18	−0.03 ± 0.40 **^1^**	0.23 ± 0.39 **^1^**	1.19	0.698	**<0.001**	0.324
	Inv1	−1.28 ± 24 **^2^**	−1.28 ± 0.18 **^2^**	1.00			
	L12	0.00 ± 0.61 **^a,1^**	1.24 ± 0.37 ^**b**^	2.38			
*Lxra*	L18	0.52 ± 0.17 **^1-2^**	0.44 ± 0.27	0.95	**0.024**	0.053	0.086
	Inv1	1.08 ± 0.07 **^2^**	1.00 ± 0.13	0.95			
Protein synthesis regulatory factors							
	L12	0.00 ± 0.48 **^1^**	0.59 ± 0.24 **^1^**	1.51			
*Akt1*	L18	−0.02 ± 0.08 **^1^**	−0.10 ± 0.18 **^2^**	0.94	0.209	**<0.001**	0.128
	Inv1	−0.81 ± 0.05 **^2^**	−0.65 ± 0.05 **^2^**	1.05			
	L12	0.00 ± 0.56 **^a^**	0.88 ± 0.23 **^b,1^**	1.84			
*Mtor*	L18	0.34 ± 0.13	0.09 ± 0.27 **^2^**	0.84	**0.040**	0.189	0.223
	Inv1	0.06 ± 0.05	0.06 ± 0.04 **^2^**	1.00			
	L12	0.00 ± 0.51	0.78 ± 0.34 **^1^**	1.71			
*Stat5*	L18	0.17 ± 0.13	0.09 ± 0.26 **^1,2^**	0.94	0.164	**0.004**	0.212
	Inv1	−0.36 ± 0.08	−0.35 ± 0.13 **^2^**	1.01			

Log2 (fold change) corresponded to −ΔΔCq values calculated with mean of 3 housekeeping genes (*Actb*, *Uxt*, *Rps9*) and with CTL at L12 as a reference group. Fold change correspond to 2^−ΔΔCq^ values and are calculated each day for FEN diet with CTL of the day as a reference. Log2 (fold change) values are mean ± SEM, *n* = 5–6 at L12 and L18 and 10–11 at Inv1. Log2 (fold change) values were analyzed with 2-way ANOVA (*p*-values of diet, period and interaction effects reported in the last columns) followed by Sidak’s *post*-*hoc* test for diet effect and Tukey’s *post*-*hoc* test for period effect. Different letters and numbers represent significant difference (*p* < 0.05) between diets for each period and between periods for each diet, respectively. Green boxes represent significant overexpression in FEN group at a given day. CTL: control diet group and FEN: fenugreek-supplemented diet group throughout the lactation period. L12 and L18: post-natal day of lactation 12 and 18, InV1: first day of involution. The values in bold correspond to significant *p*-values (<0.05).

**Table 5:** Effect of fenugreek on relative expression of genes of lactogenic hormone receptors in mammary gland along lactation period.

		Log2 (Fold Change) CTL at L12 as Reference		Fold Change CTL as Reference	2-Way ANOVA		
Genes	Period						
		CTL	FEN		Inter	Period	Diet
Lactogenic hormone receptors							
	L12 (*n* = 6)	0.00 ± 0.49 **^1^**	0.40 ± 0.26 **^1^**	1.32			
*Prlr*	L18 (*n* = 6)	−0.32 ± 0.30 **^1^**	−0.11 ± 0.49 **^1^**	1.15	0.499	**<0.001**	0.642
	Inv1 (*n* = 11)	−1.84 ± 0.16 **^2^**	−2.09 ± 0.22 **^2^**	0.84			
	L12	0.00 ± 0.64 **^1^**	0.84 ± 0.32 **^1^**	1.79			
*Insr*	L18	2.29 ± 0.19 **^2^**	2.35 ± 0.18 **^2^**	1.04	0.283	**<0.001**	0.082
	Inv1	−0.85 ± 0.09 **^3^**	−0.67 ± 0.11 **^3^**	1.13			
	L12	0.00 ± 0.62 **^1^**	0.79 ± 0.69 **^1^**	1.72			
*Oxtr*	L18	−0.52 ± 0.37 **^1^**	−0.58 ± 0.87 **^1^**	0.96	0.649	**0.025**	0.557
	Inv1	0.75 ± 0.22 **^1^**	0.73 ± 0.26 **^1^**	0.99			
	L12	0.00 ± 0.56	0.89 ± 0.43	1.86			
*Ghr*	L18	−0.14 ±0.23	0.34 ± 0.43	1.40	0.286	0.405	0.097
	Inv1	0.07 ±0.18	0.01 ± 0.18	0.96			
	L12	0.00 ± 0.59 **^a,1^**	1.37 ± 0.52 **^b,1,2^**	2.60			
*Igf1r*	L18	0.89 ± 0.11 **^1^**	0.96 ± 0.38 **^1^**	1.05	0.057	**<0.001**	**0.033**
	Inv1	1.87 ± 0.19 **^2^**	1.97 ± 0.10 **^2^**	1.07			
	L12	0.00 ± 0.54 **^1^**	0.34 ± 0.18 **^1^**	1.27			
*Esr1*	L18	1.57 ± 0.14 **^2^**	1.09 ± 0.32 **^1^**	0.72	0.204	**<0.001**	0.547
	Inv1	−2.02 ± 0.24 **^3^**	−1.44 ±0.17 **^2^**	1.49			

Log2 (fold change) corresponded to −ΔΔCq values calculated with mean of 3 housekeeping genes (*Actb*, *Uxt*, *Rps9*) and with CTL at L12 as a reference group. Fold change correspond to 2^−ΔΔCq^ values and are calculated each day for FEN diet with CTL of the day as a reference. Log2 (fold change) values are mean ± SEM, *n* = 5–6 at L12 and L18 and 10–11 at Inv1. Log2 (fold change) values were analyzed with 2-way ANOVA (*p*-values of diet, period and interaction effects reported in the last columns) followed by Sidak’s *post*-*hoc* test for diet effect and Tukey’s *post*-*hoc* test for period effect. Different letters and numbers represent significant difference (*p* < 0.05) between diets for each period and between periods for each diet, respectively. Green boxes represent significant overexpression in FEN group compared to CTL group at a given day. CTL: control diet group and FEN: fenugreek-supplemented diet group throughout the lactation period. L12 and L18: post-natal day of lactation 12 and 18, InV1: first day of involution. The values in bold correspond to significant *p*-values (<0.05).

**Table 6:** Effect of fenugreek on relative expression of lactogenic hormone-related genes in pituitary gland along lactation period.

		Log2 (Fold Change) CTL at L12 as Reference		Fold Change CTL as Reference	2-Way ANOVA		
Genes	Period						
		CTL	FEN		Inter	Period	Diet
Pituitary lactogenic hormones							
	L12 (*n* = 5)	0.00 ± 0.06 **^1^**	−0.15 ± 0.20 **^1^**	0.90			
*Prl*	L18 (*n* = 5)	−0.14 ± 0.15 **^1^**	−0.20 ± 0.11 **^1^**	0.95	0.291	**<0.001**	0.652
	Inv1 (*n* = 10)	−1.18 ± 0.06 **^2^**	−0.93 ± 0.14 **^2^**	1.19			
	L12	0.00 ± 0.25	−0.34 ± 0.05 **^1^**	0.79			
*Gh1*	L18	0.02 ± 0.19	0.21 ± 0.19 **^2^**	1.14	0.143	0.110	0.925
	Inv1	−0.24 ± 0.09	−0.06 ± 0.10 **^1-2^**	1.14			
	L12	0.00 ± 0.48	0.70 ± 0.12	1.62			
*Oxt*	L18	0.16 ± 0.44 **^a^**	1.08 ± 0.10 ^**b**^	1.88	**0.050**	0.469	**0.007**
	Inv1	0.42 ± 0.08	0.38 ± 0.12	0.97			
Receptors of lactogenic hormone activators							
	L12	0.00 ± 0.05 **^1^**	−0.13 ± 0.06 **^1^**	0.91			
*Drd2*	L18	−0.39 ± 0.04 **^2^**	−0.45 ± 0.07 **^2^**	0.96	**0.040**	**<0.001**	0.984
	Inv1	−0.46 ± 0.06 **^a,2^**	−0.27 ± 0.06 **^b,1,2^**	1.14			
	L12	0.00 ± 0.16 **^1^**	0.42 ± 0.21 **^1^**	1.34			
*Trhr*	L18	0.78 ± 0.10 **^2^**	0.99 ± 0.10 **^2^**	1.15	0.551	**<0.001**	**0.035**
	Inv1	0.55 ± 0.13 **^2^**	0.67 ± 0.07 **^1-2^**	1.09			
	L12	0.00 ± 0.25	0.35 ± 0.11 **^1^**	1.27			
*Vipr2*	L18	0.65 ± 0.16	1.07 ± 0.07 **^2^**	1.34	0.375	**<0.001**	0.061
	Inv1	0.22 ± 0.15	0.25 ± 0.14 **^1^**	1.02			
	L12	0.00 ± 0.16 **^1^**	−0.65 ± 0.44 **^1^**	0.64			
*Ghrhr*	L18	0.46 ± 0.33 **^1^**	1.20 ± 0.21 **^2^**	1.68	0.069	**<0.001**	0.382
	Inv1	−2.31 ± 0.15 **^2^**	−1.77 ± 0.27 **^3^**	1.45			
	L12	0.00 ± 0.08	0.11 ± 0.09	1.08			
*Ar*	L18	0.27 ± 0.08	0.18 ± 0.09	0.94	0.420	0.152	0.565
	Inv1	0.11 ± 0.04	0.19 ± 0.07	1.06			
	L12	0.00 ± 0.14 **^1^**	0.26 ± 0.35 **^1^**	1.20			
*Esr1*	L18	0.56 ± 0.12 **^1-2^**	0.69 ± 0.09 **^1-2^**	1.09	0.508	**<0.001**	0.823
	Inv1	1.35 ± 0.16 **^2^**	1.10 ± 0.27 **^2^**	0.84			
Estrogenic effect biomarker							
	L12	0.00 ± 0.29	−0.09 ± 0.35 **^1^**	0.94			
*C3*	L18	0.24 ± 0.18 **^a^**	1.31 ± 0.15 **^b,2^**	2.10	**0.017**	**0.006**	**0.006**
	Inv1	0.61 ± 0.26	1.00 ± 0.13 **^2^**	1.31			

Log2 (fold change) corresponded to −ΔΔCq values calculated with mean of 3 housekeeping genes (*Actb*, *Uxt*, *Rps9*) and with CTL at L12 as a reference group. Fold change correspond to 2^−ΔΔCq^ values and are calculated each day for FEN diet with CTL of the day as a reference. Log2 (fold change) values are mean ± SEM, *n* = 4–5 at L12 and L18 and 9–10 at Inv1 (for ESR1 *n* = 4 for CTL at L12). Log2 (fold change) values were analyzed with 2-way ANOVA (*p*-values of diet, period and interaction effects reported in the last columns) followed by Sidak’s *post*-*hoc* test for diet effect and Tukey’s *post*-*hoc* test for period effect. Different letters and numbers represent significant difference (*p* < 0.05) between diets for each period and between periods for each diet, respectively. Green boxes represent significant overexpression in FEN group compared to CTL group at a given day. CTL: control diet group and FEN: fenugreek-supplemented diet group throughout the lactation period. L12 and L18: post-natal day of lactation 12 and 18, InV1: first day of involution. The values in bold correspond to significant *p*-values (<0.05).

## Supplementary Materials

The following are available online at https://www.mdpi.com/2073-4425/11/10/1208/s1, Figure S1: PLS-DA score plot on non-normalized mammary gene expression separating groups according to the diet and the lactation period. Figure S2: Pearson correlation table between all mammary gland genes’ expressions on row data. Figure S3: Pearson correlation table between all mammary gland genes’ expressions on data normalized by the period of lactation. Figure S4: Food intake of dams supplemented or not with 1 g/kg/day fenugreek during lactation. Table S1: Validation of RNA extraction and primer design.

## Abbreviations

GH: growth hormone; IGF-1: insulin-like growth factor 1; GHR: GH receptor; IGF1R: IGF-1 receptor; INSR: insulin receptor; PCA: principal component analysis; PLS-DA: partial least square discriminant analysis; AKT: serine/threonine kinase; mTOR: mechanistic target of rapamycin.

## Appendix A. List of the Primers’ Sequences

**Table A1:** List of the primers’ sequences used to measure each gene by qPCR with the amplicon size and the efficiency.

Gene Name	Gene Symbol	Function	Accession Number	Primer Sequences (5′ to 3′)	Amplicon (pb)	Efficiency (%)
Lipoprotein lipase	*Lpl*	Triglyceride hydrolysis and mediation of lipoprotein uptakes	NM_012598	F: AACTGCCACTTCAACCACAGC R: CATACATTCCTGTCACCGTCCA	70	95.8
Fatty acid synthase	*Fasn*	Fatty acid biosynthesis, mainly palmitate and long chain fatty acid synthesis	NM_017332	F: CGCCGTGGTGCTGGAGATTG R: CTTGCCGAGGTTGGTGAGGAAG	142	98.3
Acetyl-CoA carboxylase α	*Acaca*, *Acc1*	Catalyze the rate limiting step in fatty acid biosynthesis: malonyl-CoA synthesis	NM_022193	F: CGATTCCCATCCGCCTCTTCC R: GGTCCCTGCTTGTCTCCATACG	127	92.3
Acetyl-CoA carboxylase β	*Acacb*	Fatty acid biosynthesis, involved in inhibition of fatty acid oxidation	NM_053922	F: GACGCCAGCAACATCACTTCG R: GCCTCTCGGTCCACCTTACG	107	85.5
Fatty acid binding protein 3	*Fabp3*	Uptake, intracellular metabolism and transport of fatty acids	NM_024162	F: ACAGGAAGGTCAAGTCGGTC R: ATGGGTGAGAGTCAGGATGAG	127	93.3
Fatty acid binding protein 4	*Fabp4*	Lipid binding protein involved in fatty acid trafficking, main adipocyte FABP	NM_053365	F: TGTGGGGACCTGGAAACTCGT R: CGAAGCCAACTCCCACTTCTTT	71	92.9
Stearoyl-CoA desaturase	*Scd*, *Scd1*	Fatty acid desaturation, biosynthesis of oleic acid.	NM_139192	F: GTTGGGTGCCTTATCGCTTTCC R: CTCCAGCCAGCCTCTTGTCTA	114	97.8
Diacylglycerol O-acyltransferase 1	*Dgat1*	Catalyzes the synthesis of triglyceride from diglyceride and acyl-CoA	NM_053437	F: GGCATCATACTCCATCATCTTC R: CCCACTGACCTTCTTCCC	115	97.1
Diacylglycerol O-acyltransferase 2	*Dgat2*	Catalyzes the synthesis of triglyceride from diglyceride and acyl-CoA	NM_001012345	F: GGTCATCTCAGTCCTACAG R: CTATCAGCCAGCAGTCAG	101	88.6
Perilipin 2	*Plin2*	Coating of intracellular lipid droplets and milk fat globules, involved in milk lipid secretion.	NM_001007144	F: CTTCTTCATTGACCTGCGAC R: CACCCTTACCTTAAGTTTCTCCT	108	97.1
Solute carrier family 2, facilitated glucose transporter member 1	*Slc2a1, Glut1*	One of major glucose transporters, located in the cell membrane	NM_138827	F: CCAATATGTGGAGCAACTGTG R: GGAAGCGATCTCATCGAAGG	129	108.2
Phosphoglucomutase 1	*Pgm1*	Interconvert glucose-1-phosphate and glucose-6-phosphate	NM_017033	F: CCCTTCACAGTGGAGATCGT R: GTCGATGCGGATCTTCAGTC	122	96.1
UDP-glucose pyrophosphorylase 2	*Ugp2*	Convert glucose-1-phosphate to UDP-glucose	NM_001024743	F: CGGAAGATTCGATTCAACCC R: GTTCCCAAACCACCATTGAG	111	93.0
β-1,4- galactosyltransferase 1	*B4galt1*	Catalytic subunit of lactose synthase complex, convert UDP-galactose and glucose into lactose	NM_053287	F: CGCTTTGTGTTCAGTGATGTGG R: AACGTAAGGCAGGCTAAACC	122	100.7
Lacatlbumin α	*Lalba*	Regulatory subunit of lactose synthase complex, one of the main milk proteins	NM_012594	F: CTTGCTTGAATGGACCTGTG R: GCTACTCTTGCACCAATTTCTG	126	92.7
Casein β	*Csn2*	Principal milk protein and primary source of essential amino acids for sucking young	NM_017120	F: AAACATCCAGCCTATTGCTC R: CATCTGTTTGTGCTTGGGAA	118	96.6
Whey acidic protein	*Wap*	Whey protein associated with mammary gland, a major constituent of milk	NM_053751	F: CATGTCTTCAACTCAGTTCAGTCC R: CATGTCATTCTGGGCACACTC	104	95.8
Solute carrier family 7 member 5	*Slc7a5*	Amino acid transporter, mediate the uptake of large neutral amino acids (branch-chain, aromatic)	NM_017353	F: GGGAAAGGACATAGGACAAGG R: TCAGATAGTTCCATCCTCCGT	136	94.5
Aquaporin 1	*Aqp1*	Integral membrane protein, allow the passive transport of water along osmotic gradient.	NM_012778	F: GGCATTGAGATCATTGGCAC R: GTGTAGTCAATGGCCAGCAG	142	97.8
Aquaporin 3	*Aqp3*	Passive water transporter located in the basolateral cell membrane	NM_031703	F: GGGCTCTACTATGATGCAATCTG R: GCTGCTGTGCCTATGAACTG	148	95.5
Carnitine palmitoyltransferase 1A	*Cpt1a*	Initiate β-oxidation by translocating long chain fatty acids across mitochondrial inner membrane	NM_031559	F: TGCCTGCCAGTTCCATTAAGC R: GTCTCACTCCTCTTGCCAACAG	143	90.1
Acyl-CoA dehydrogenase short chain	*Acads*	Catalyze the first step of mitochondrial fatty acid β-oxidation	NM_022512	F: CGGCAGAACAAGGGTATCAG R: AAGATGAGGTTAGCTGTAGATGAG	115	99.1
Pyruvate dehydrogenase E1 subunit α 1	*Pdha1*	Subunit of the pyruvate dehydrogenase complex that convert pyruvate to acetyl-CoA and CO_2_	NM_001004072	F: CACGGACCATCTCATCACTG R: CTCCTCTTCGTCCTGTTAATTCTG	100	91.2
Citrate synthase	*Cs*	Krebs cycle enzyme that catalyzes the synthesis of citrate from oxaloacetate and acetyl-CoA	NM_130755	F: GCTATAGTATCCCTGAGTGCCA R: AGAGCCAAGAGACCTGTTCC	128	91.1
Cytochrome c oxidase I, mitochondrial	*Mtco1*, *mtCox1*	Subunit of the cytochrome c oxidase, catalyzes reduction of oxygen to water for oxidative phosphorylation	YP_665631	F: GCCTAGATGTAGACACCCGAG R: TATTTCCTCCATGTAGTGTAGCGA	107	97.0
ATP synthase F1 subunit α	*Atp5f1a*	Subunit of the mitochondrial ATP synthase, catalyzes the ATP synthesis during oxidative phosphorylation	NM_023093	F: AGCGTTTCAATGATGGGACTG R: GTACTTCATGGCATCTGCGTC	124	94.3
Sterol regulatory element binding transcription factor 1	*Srebf1*	Nuclear transcription factor that bind to the sterol regulatory element 1, regulates the transcription of genes important for sterol biosynthesis and lipogenesis	NM_001276707	F: GCAGCTGATGGAGACAGGGA R: CGACAGCGTCAGAACAGCTA	76	99.3
Peroxisome proliferator activated receptor γ (PPARγ)	*Pparg*	Transcription factor that regulate expression of genes involved in lipid metabolism	NM_001145366	F: CCGTTCACAAGAGCTGACCC R: TCGCACTTTGGTATTCTTGGAGC	73	94.7
Thyroid hormone responsive	*Thrsp*, *Spot14*	Play a role in regulation of lipogenesis and specially triglycerides with medium-chain fatty acids.	NM_012703	F: CACATCCTTACCCACCTGAC R: CGTGTAAAGTGATCTTCCATAGAG	109	88.5
Nuclear receptor subfamily 1, group H, member 3 (LXRα)	*Nr1h3*, *Lxra*	Nuclear oxysterol receptor that regulate inflammation and homeostasis of lipids and cholesterol	NM_031627	F: CAGAGCCTACAGAACTTCGT R: CAGCTCAGCACATTGTAATGG	123	101.0
AKT serine/threonine kinase 1 (AKT)	*Akt1*	Key signaling pathway protein notably linked to mTOR signaling, regulate metabolism, proliferation	NM_033230	F: CTACTATGCCATGAAGATCCTC R: CTACTATGCCATGAAGATCCTC	124	93.9
Mechanistic target of rapamycin kinase (mTOR)	*Mtor*	Central regulator of cell metabolism, growth and survival, mediate nutrient and energy signal, key regulator of mRNA translation	NM_019906	F: ACCAATTATACTCGCTCCCTG R: CATAGCAACCTCAAAGCAGTC	147	90.9
Signal transducer and activator of transcription 5A (STAT5)	*Stat5a*, *Stat5*	Involved in signal transduction and transcription activation in response to cytokine, growth factors and prolactin	NM_017064	F: AGAACACCCGCAATGATTACAG R: GTGACATGTTTCTGAAGTGGG	129	97.8
Prolactin receptor	*Prlr*	Prolactin binding and signaling mainly through JAK2/STAT5 and Akt/mTOR pathways	NM_001034111	F: GAAATGCCAAATGACTTCACCT R: TATAGCCCTTCAAAGCCACTG	111	91.5
Insulin receptor	*Insr*	Insulin binding and signaling mainly through AKT/mTOR and MAPK pathways	NM_017071	F: TTCATTCAGGAAGACCTTCGA R: CAGGCCAGAGATGACAAGTGAC	259	95.4
Oxytocin receptor	*Oxtr*	G-protein coupled receptor binding oxytocin, activate PIP3-Ca^2+^ pathway	NM_012871	F: TTCATTCAGGAAGACCTTCGA R: GAGTTCGTGGAAGAGGTGAC	146	88.3
Growth hormone receptor	*Ghr*	Growth hormone binding and signaling mainly through JAK2/STAT5 pathway	NM_017094	F: TTCTTCGTGCAGATGTGGAG R: ATGGAAACCGGAAATCTTCTTCAC	115	102.1
Insulin-like growth factor 1 receptor	*Igf1r*	IGF-1 binding and signaling mainly through AKT/mTOR and MAPK pathways.	NM_052807	F: CCATAGAAAGAGGAATAACAGCAG R: TACCTCCCATTCATCAGGCA	108	88.9
Estrogen receptor 1	*Esr1*, *Esrα*	Nuclear receptor binding estrogens, act as a regulator of DNA transcription	NM_012689	F: CCACGATCAAGTTCACCTTCTG R: CACATTTACCTTGATTCCTGTCCA	136	86.8 (MG) 92.1 (PG)
Complement C3	*C3*	Involved in innate immune response; central role in activating the complement system	NM_000064	F: CTTCTTCATTGACCTGCGAC R: CACCCTTACCTTAAGTTTCTCCT	122	94.6 (MG) 98.4 (PG)
Superoxide dismutase 1	*Sod1*	Convert superoxide to hydrogen peroxide and oxygen, involved in response to oxidative stress	NM_017050	F: TACACAAGGCTGTACCACTG R: CACACGATCTTCAATGGACAC	150	101.4
Catalase	*Cat*	Key antioxidant enzyme that convert hydrogen peroxide to water and oxygen, limit oxidative stress	NM_012520	F: CATAGCCAGAAGAGAAACCC R:GAACAAGAAAGTAACCTGATGGAG	104	93.7
Glutathione peroxidase 1	*Gpx1*	Catalyze the reduction of hydroperoxides and H_2_O_2_ by glutathione to protect cells against oxidative damages	NM_030826	F: GTTCGGACATCAGGAGAATGG R: GAAGGTAAAGAGCGGGTGAG	144	95.7
Prolactin	*Prl*	Pituitary hormone that primarily promote lactation. Play a role as growth regulator and in cell survival	NM_012629	F: AAACAGTATGTCCAAGATCGTGAG R: ACTTCCGGAGGGACTTTCTG	112	99.8
Gh	*Gh1*	Pituitary hormone playing major role in growth control. Stimulate IGF1-1 secretion by the liver	NM_001034848	F: ACCTACAAAGAGTTCGAGCGT R: GAAGCAATTCCATGTCAGTTCTC	147	94.2
Oxytocin/neurophysin I prepropeptide	*Oxt*	Hormone involved in contraction of smooth muscle during parturition and lactation	NM_012996	F: GGATATGCGCAAGTGTCTTCC R: GAGGGCAGGTAGTTCTCCTC	140	106.8
Dopamine receptor D2	*Drd2*	G-protein coupled receptor of dopamine which inhibit adenylyl-cyclase and notably prolactin secretion	NM_012547	F: CCACTCAAGGGCAACTGTACC R: ATGGGGCTATACCGGGTCC	179	91.5
Thyrotropin releasing hormone receptor	*Trhr*	G-protein coupled receptor of TRH that activate PIP3-Ca^2+^ pathway, promote release of prolactin	NM_013047	F: CAGACCGCTTTAGCACAGAG R: ACTGGGTCCATTCTTCTCGG	112	90.1
Vasoactive intestinal peptide receptor 2	*Vipr2*	G-protein coupled receptor of VIP which activate adenylyl-cyclase and notably prolactin secretion	NM_017238	F: GCAGCCAAATGGAGAATCAC R: TTTGCTTATGTTTCCTGGTCTG	145	104.0
Growth hormone releasing hormone receptor	*Ghrhr*	G-protein coupled receptor of GHRH that activate adenylyl-cyclase and stimulate GH transcription and secretion	NM_012850	F: GCTGTTTGCTACTTTCATCCT R: CCTTGCACAGAATAGTGGAC	109	97.9
Adrogen receptor	*Ar*	Steroid hormone activated transcription factor, involved in cell proliferation and differentiation	NM_012502	F: CTCTGCCTCTGAAGTGTGGT R: TACTGTCCAAACGCATGTCC	136	91.5
Actin β	*Actb*	Highly conserved proteins that are involved in cell motility, structure, integrity and signaling	NM_031144	F: CTATCGGCAATGAGCGGTTCC R: GCACTGTGTTGGCATAGAGGTC	150	93.2 (PG)
Ubiquitously expressed, prefoldin-like chaperone	*Uxt*	Cofactor that modulates AR-dependent transcription, and also plays a critical role in TNF-induced apoptosis	NM_001006982	F: ATTGACCGAAAGAGTTCCCT R: GTAGTTCTCTAAGTCCCTCTAGCA	108	91.3 (MG) 87.2 (PG)
Ribosomal protein S9	*Rps9*	One of 40S ribosomal subunit protein	NM_031108	F: GCTAAAGTTGATTGGAGAGTATGG R: AGAGCGTTGCCTTCAAACAG	146	99.0 (MG) 89.5 (PG)
Valosin-containing protein	*Vcp*	ATP binding protein involved in vesicle transport and fusion and endoplasmic reticulum function	NM_053864	F: TCAAGCGAGAGGATGAGGAG R: TCACACCAATTGCCTTAAAGAG	140	92.3

Official name of each gene with official symbol given by NCBI gene (first symbol) as well as the most common symbol used if different. Functions were given by NCBI gene or GeneCards. Primers of *Actb*, *Lpl*, *Fasn*, *Acaca*, *Acacb*, *Scd1*, *Dgat1*, *Dgat2*, *Fabp4*, *Cpt1a*, *Srebf1*, *Pparg*, *Akt1*, *mTOR*, *Insr* and *Drd2* were already designed and validated in the lab, whereas other primers were designed using PerlPrimer. All primers were tested for efficiency and specificity (RT-) in mammary gland or/and pituitary gland. MG and PG: efficiency measured in mammary gland and pituitary gland respectively, if genes’ expression was measured in both tissues.
