# Supplementary material for: Fenugreek Stimulates the Expression of Genes Involved in Milk Synthesis and Milk Flow through Modulation of Insulin/GH/IGF-1 Axis and Oxytocin Secretion
Source: Genes (Basel). 2020 Oct 16;11(10):1208. doi: 10.3390/genes11101208 (PMC7602737; doi:10.3390/genes11101208)
Supplement: Supplementary file 1 [file genes-11-01208-s001.pdf]

Supplementary Materials

Figure S1: Pearson correlation table between all mammary gland genes expressions on row data

	Lpl	Fasn	Acaca	Acacb	Fabp3	Fabp4	Scd1	Dgat1	Dgat2	Plin2	Glut1	Pgm1	Ugp2	B4galt1	Lalba	Csn2	Wap	Slc7a5	Aqp1	Aqp3	Cpt1a	Acads	Pdha1	Cs	Mtco1	Atp5f1a	Srebf1	Pparg	Spot14	Lxra	Akt1	Mtor	Stat5	Insr	Prlr	Oxtr	Esr1	Ghr	Igf1r	Sod1	Cat	Gpx1
Lpl		0.97	0.98	0.88	0.95	-0.17	0.51	0.73	-0.49	0.59	0.86	0.04	0.92	0.94	0.81	0.85	0.74	0.79	0.65	0.74	-0.69	-0.36	0.92	0.89	0.81	0.69	0.73	0.77	0.79	-0.15	0.76	0.38	0.62	0.81	0.89	-0.12	0.86	0.31	-0.46	0.08	-0.08	0.73
Fasn	0.97		0.99	0.84	0.95	-0.18	0.44	0.70	-0.49	0.56	0.89	0.08	0.92	0.95	0.81	0.86	0.76	0.81	0.62	0.73	-0.71	-0.38	0.90	0.89	0.78	0.66	0.75	0.74	0.79	-0.19	0.74	0.35	0.60	0.77	0.87	-0.16	0.87	0.26	-0.49	0.11	-0.05	0.67
Acaca	0.98	0.99		0.86	0.96	-0.14	0.48	0.67	-0.51	0.53	0.90	0.11	0.94	0.96	0.78	0.84	0.74	0.79	0.59	0.69	-0.69	-0.37	0.91	0.90	0.80	0.71	0.79	0.76	0.77	-0.17	0.77	0.37	0.62	0.74	0.88	-0.12	0.84	0.30	-0.48	0.11	-0.04	0.70
Acacb	0.88	0.84	0.86		0.79	-0.09	0.56	0.55	-0.39	0.48	0.74	0.17	0.82	0.78	0.74	0.76	0.66	0.60	0.52	0.69	-0.46	-0.13	0.80	0.80	0.73	0.70	0.65	0.67	0.79	0.05	0.66	0.41	0.66	0.71	0.85	0.06	0.75	0.45	-0.26	0.22	0.03	0.68
Fabp3	0.95	0.95	0.96	0.79		-0.22	0.40	0.71	-0.61	0.62	0.90	0.03	0.92	0.97	0.85	0.88	0.83	0.89	0.62	0.67	-0.69	-0.34	0.90	0.89	0.76	0.66	0.70	0.73	0.71	-0.19	0.78	0.37	0.55	0.76	0.79	-0.17	0.87	0.23	-0.46	-0.01	-0.15	0.57
Fabp4	-0.17	-0.18	-0.14	-0.09	-0.22		-0.09	-0.58	0.11	-0.56	-0.11	0.56	0.08	-0.34	-0.30	-0.28	-0.31	-0.45	-0.17	-0.39	0.51	0.48	-0.19	-0.07	-0.02	0.19	0.31	0.29	0.06	0.47	0.11	0.34	0.22	-0.51	0.10	0.67	-0.28	0.50	0.28	0.60	0.49	-0.10
Scd1	0.51	0.44	0.48	0.56	0.40	-0.09		0.40	-0.06	0.35	0.30	-0.10	0.37	0.46	0.36	0.38	0.24	0.33	0.34	0.45	-0.27	-0.12	0.46	0.44	0.45	0.39	0.29	0.34	0.45	0.07	0.39	0.19	0.37	0.48	0.42	0.02	0.45	0.33	-0.05	-0.20	-0.19	0.47
Dgat1	0.73	0.70	0.67	0.55	0.71	-0.58	0.40		-0.34	0.90	0.49	-0.47	0.50	0.78	0.77	0.79	0.73	0.79	0.70	0.82	-0.65	-0.42	0.71	0.62	0.63	0.30	0.20	0.40	0.49	-0.27	0.49	0.13	0.32	0.88	0.51	-0.45	0.73	-0.07	-0.35	-0.39	-0.39	0.36
Dgat2	-0.49	-0.49	-0.51	-0.39	-0.61	0.11	-0.06	-0.34		-0.37	-0.56	-0.06	-0.60	-0.53	-0.60	-0.61	-0.65	-0.51	-0.48	-0.23	0.37	0.07	-0.56	-0.57	-0.46	-0.45	-0.45	-0.45	-0.38	0.04	-0.59	-0.38	-0.41	-0.39	-0.45	-0.07	-0.55	-0.11	0.22	0.11	0.10	-0.24
Plin2	0.59	0.56	0.53	0.48	0.62	-0.56	0.35	0.90	-0.37		0.34	-0.49	0.41	0.67	0.81	0.79	0.76	0.73	0.75	0.74	-0.50	-0.25	0.66	0.56	0.58	0.30	0.06	0.33	0.42	-0.10	0.47	0.24	0.31	0.85	0.41	-0.38	0.73	-0.02	-0.18	-0.44	-0.33	0.19
Glut1	0.86	0.89	0.90	0.74	0.90	-0.11	0.30	0.49	-0.56	0.34		0.21	0.86	0.86	0.67	0.72	0.68	0.77	0.39	0.50	-0.68	-0.42	0.76	0.79	0.60	0.58	0.72	0.70	0.60	-0.29	0.60	0.24	0.42	0.55	0.72	-0.12	0.71	0.21	-0.53	0.20	0.00	0.56
Pgm1	0.04	0.08	0.11	0.17	0.03	0.56	-0.10	-0.47	-0.06	-0.49	0.21		0.30	-0.05	-0.15	-0.10	-0.10	-0.18	-0.17	-0.27	0.18	0.22	0.00	0.11	0.01	0.26	0.46	0.27	0.16	0.20	0.15	0.25	0.24	-0.37	0.23	0.27	-0.10	0.25	-0.04	0.49	0.42	0.06
Ugp2	0.92	0.92	0.94	0.82	0.92	0.08	0.37	0.50	-0.60	0.41	0.86	0.30		0.87	0.73	0.79	0.71	0.69	0.57	0.57	-0.53	-0.18	0.87	0.89	0.79	0.78	0.88	0.83	0.82	-0.02	0.84	0.52	0.72	0.61	0.91	0.04	0.78	0.40	-0.39	0.23	0.06	0.64
B4galt1	0.94	0.95	0.96	0.78	0.97	-0.34	0.46	0.78	-0.53	0.67	0.86	-0.05	0.87		0.82	0.87	0.80	0.90	0.61	0.73	-0.73	-0.40	0.90	0.88	0.76	0.62	0.64	0.66	0.68	-0.22	0.73	0.31	0.52	0.82	0.77	-0.27	0.85	0.18	-0.47	-0.10	-0.19	0.60
Lalba	0.81	0.81	0.78	0.74	0.85	-0.30	0.36	0.77	-0.60	0.81	0.67	-0.15	0.73	0.82		0.98	0.96	0.84	0.76	0.77	-0.57	-0.21	0.83	0.79	0.70	0.55	0.42	0.60	0.71	-0.05	0.69	0.41	0.55	0.83	0.70	-0.06	0.92	0.23	-0.28	-0.05	-0.18	0.30
Csn2	0.85	0.86	0.84	0.76	0.88	-0.28	0.38	0.79	-0.61	0.79	0.72	-0.10	0.79	0.87	0.98		0.95	0.85	0.78	0.79	-0.62	-0.26	0.86	0.83	0.75	0.58	0.49	0.64	0.73	-0.08	0.72	0.42	0.58	0.83	0.75	-0.10	0.93	0.23	-0.33	-0.04	-0.15	0.36
Wap	0.74	0.76	0.74	0.66	0.83	-0.31	0.24	0.73	-0.65	0.76	0.68	-0.10	0.71	0.80	0.96	0.95		0.83	0.71	0.71	-0.58	-0.25	0.78	0.75	0.64	0.50	0.40	0.57	0.62	-0.11	0.65	0.36	0.49	0.74	0.64	-0.12	0.85	0.13	-0.32	-0.02	-0.16	0.18
Slc7a5	0.79	0.81	0.79	0.60	0.89	-0.45	0.33	0.79	-0.51	0.73	0.77	-0.18	0.69	0.90	0.84	0.85	0.83		0.57	0.68	-0.70	-0.41	0.77	0.74	0.60	0.42	0.39	0.53	0.52	-0.27	0.58	0.21	0.34	0.76	0.55	-0.30	0.80	0.06	-0.41	-0.23	-0.31	0.32
Aqp1	0.65	0.62	0.59	0.52	0.62	-0.17	0.34	0.70	-0.48	0.75	0.39	-0.17	0.57	0.61	0.76	0.78	0.71	0.57		0.68	-0.35	-0.03	0.72	0.68	0.76	0.50	0.33	0.60	0.61	0.21	0.65	0.59	0.61	0.77	0.63	-0.01	0.74	0.29	-0.05	-0.16	-0.01	0.33
Aqp3	0.74	0.73	0.69	0.69	0.67	-0.39	0.45	0.82	-0.23	0.74	0.50	-0.27	0.57	0.73	0.77	0.79	0.71	0.68	0.68		-0.43	-0.20	0.77	0.73	0.72	0.52	0.34	0.55	0.68	0.03	0.58	0.34	0.56	0.88	0.65	-0.14	0.74	0.29	-0.08	-0.06	-0.15	0.42
Cpt1a	-0.69	-0.71	-0.69	-0.46	-0.69	0.51	-0.27	-0.65	0.37	-0.50	-0.68	0.18	-0.53	-0.73	-0.57	-0.62	-0.58	-0.70	-0.35	-0.43		0.87	-0.55	-0.45	-0.30	-0.10	-0.33	-0.23	-0.35	0.72	-0.24	0.25	0.00	-0.55	-0.46	0.55	-0.64	0.32	0.87	0.10	0.24	-0.45
Acads	-0.36	-0.38	-0.37	-0.13	-0.34	0.48	-0.12	-0.42	0.07	-0.25	-0.42	0.22	-0.18	-0.40	-0.21	-0.26	-0.25	-0.41	-0.03	-0.20	0.87		-0.18	-0.07	0.04	0.27	-0.05	0.05	0.03	0.89	0.18	0.59	0.39	-0.22	-0.10	0.66	-0.27	0.55	0.86	0.08	0.21	-0.22
Pdha1	0.92	0.90	0.91	0.80	0.90	-0.19	0.46	0.71	-0.56	0.66	0.76	0.00	0.87	0.90	0.83	0.86	0.78	0.77	0.72	0.77	-0.55	-0.18		0.96	0.87	0.81	0.70	0.79	0.82	0.05	0.86	0.57	0.72	0.87	0.88	-0.06	0.90	0.42	-0.26	-0.01	-0.11	0.64
Cs	0.89	0.89	0.90	0.80	0.89	-0.07	0.44	0.62	-0.57	0.56	0.79	0.11	0.89	0.88	0.79	0.83	0.75	0.74	0.68	0.73	-0.45	-0.07	0.96		0.89	0.86	0.76	0.84	0.80	0.14	0.90	0.63	0.78	0.79	0.88	0.06	0.85	0.51	-0.17	0.05	0.01	0.62
Mtco1	0.81	0.78	0.80	0.73	0.76	-0.02	0.45	0.63	-0.46	0.58	0.60	0.01	0.79	0.76	0.70	0.75	0.64	0.60	0.76	0.72	-0.30	0.04	0.87	0.89		0.83	0.69	0.85	0.77	0.28	0.87	0.72	0.82	0.78	0.85	0.10	0.74	0.57	-0.04	-0.02	0.10	0.60
Atp5f1a	0.69	0.66	0.71	0.70	0.66	0.19	0.39	0.30	-0.45	0.30	0.58	0.26	0.78	0.62	0.55	0.58	0.50	0.42	0.50	0.52	-0.10	0.27	0.81	0.86	0.83		0.79	0.82	0.79	0.46	0.91	0.82	0.90	0.58	0.82	0.33	0.65	0.75	0.12	0.19	0.13	0.58
Srebf1	0.73	0.75	0.79	0.65	0.70	0.31	0.29	0.20	-0.45	0.06	0.72	0.46	0.88	0.64	0.42	0.49	0.40	0.39	0.33	0.34	-0.33	-0.05	0.70	0.76	0.69	0.79		0.76	0.75	0.06	0.79	0.51	0.72	0.34	0.84	0.20	0.53	0.50	-0.28	0.34	0.20	0.63
Pparg	0.77	0.74	0.76	0.67	0.73	0.29	0.34	0.40	-0.45	0.33	0.70	0.27	0.83	0.66	0.60	0.64	0.57	0.53	0.60	0.55	-0.23	0.05	0.79	0.84	0.85	0.82	0.76		0.73	0.30	0.81	0.73	0.74	0.56	0.82	0.23	0.64	0.66	-0.05	0.27	0.12	0.50
Spot14	0.79	0.79	0.77	0.79	0.71	0.06	0.45	0.49	-0.38	0.42	0.60	0.16	0.82	0.68	0.71	0.73	0.62	0.52	0.61	0.68	-0.35	0.03	0.82	0.80	0.77	0.79	0.75	0.73		0.21	0.80	0.62	0.83	0.67	0.90	0.19	0.78	0.55	-0.15	0.20	0.03	0.57
Lxra	-0.15	-0.19	-0.17	0.05	-0.19	0.47	0.07	-0.27	0.04	-0.10	-0.29	0.20	-0.02	-0.22	-0.05	-0.08	-0.11	-0.27	0.21	0.03	0.72	0.89	0.05	0.14	0.28	0.46	0.06	0.30	0.21		0.32	0.77	0.56	0.01	0.13	0.69	-0.05	0.76	0.84	0.15	0.27	-0.03
Akt1	0.76	0.74	0.77	0.66	0.78	0.11	0.39	0.49	-0.59	0.47	0.60	0.15	0.84	0.73	0.69	0.72	0.65	0.58	0.65	0.58	-0.24	0.18	0.86	0.90	0.87	0.91	0.79	0.81	0.80	0.32		0.77	0.88	0.66	0.83	0.23	0.74	0.59	0.00	0.02	0.01	0.52
Mtor	0.38	0.35	0.37	0.41	0.37	0.34	0.19	0.13	-0.38	0.24	0.24	0.25	0.52	0.31	0.41	0.42	0.36	0.21	0.59	0.34	0.25	0.59	0.57	0.63	0.72	0.82	0.51	0.73	0.62	0.77	0.77		0.85	0.41	0.59	0.52	0.45	0.78	0.44	0.12	0.19	0.26
Stat5	0.62	0.60	0.62	0.66	0.55	0.22	0.37	0.32	-0.41	0.31	0.42	0.24	0.72	0.52	0.55	0.58	0.49	0.34	0.61	0.56	0.00	0.39	0.72	0.78	0.82	0.90	0.72	0.74	0.83	0.56	0.88	0.85		0.56	0.83	0.45	0.62	0.73	0.23	0.21	0.18	0.50
Insr	0.81	0.77	0.74	0.71	0.76	-0.51	0.48	0.88	-0.39	0.85	0.55	-0.37	0.61	0.82	0.83	0.83	0.74	0.76	0.77	0.88	-0.55	-0.22	0.87	0.79	0.78	0.58	0.34	0.56	0.67	0.01	0.66	0.41	0.56		0.68	-0.20	0.86	0.27	-0.16	-0.24	-0.23	0.56
Prlr	0.89	0.87	0.88	0.85	0.79	0.10	0.42	0.51	-0.45	0.41	0.72	0.23	0.91	0.77	0.70	0.75	0.64	0.55	0.63	0.65	-0.46	-0.10	0.88	0.88	0.85	0.82	0.84	0.82	0.90	0.13	0.83	0.59	0.83	0.68		0.17	0.79	0.53	-0.28	0.30	0.15	0.70
Oxtr	-0.12	-0.16	-0.12	0.06	-0.17	0.67	0.02	-0.45	-0.07	-0.38	-0.12	0.27	0.04	-0.27	-0.06	-0.10	-0.12	-0.30	-0.01	-0.14	0.55	0.66	-0.06	0.06	0.10	0.33	0.20	0.23	0.19	0.69	0.23	0.52	0.45	-0.20	0.17		-0.07	0.71	0.55	0.44	0.40	-0.04
Esr1	0.86	0.87	0.84	0.75	0.87	-0.28	0.45	0.73	-0.55	0.73	0.71	-0.10	0.78	0.85	0.92	0.93	0.85	0.80	0.74	0.74	-0.64	-0.27	0.90	0.85	0.74	0.65	0.53	0.64	0.78	-0.05	0.74	0.45	0.62	0.86	0.79	-0.07		0.30	-0.32	-0.02	-0.15	0.50
Ghr	0.31	0.26	0.30	0.45	0.23	0.50	0.33	-0.07	-0.11	-0.02	0.21	0.25	0.40	0.18	0.23	0.23	0.13	0.06	0.29	0.29	0.32	0.55	0.42	0.51	0.57	0.75	0.50	0.66	0.55	0.76	0.59	0.78	0.73	0.27	0.53	0.71	0.30		0.51	0.31	0.31	0.34
Igf1r	-0.46	-0.49	-0.48	-0.26	-0.46	0.28	-0.05	-0.35	0.22	-0.18	-0.53	-0.04	-0.39	-0.47	-0.28	-0.33	-0.32	-0.41	-0.05	-0.08	0.87	0.86	-0.26	-0.17	-0.04	0.12	-0.28	-0.05	-0.15	0.84	0.00	0.44	0.23	-0.16	-0.28	0.55	-0.32	0.51		-0.04	0.10	-0.30
Sod1	0.08	0.11	0.11	0.22	-0.01	0.60	-0.20	-0.39	0.11	-0.44	0.20	0.49	0.23	-0.10	-0.05	-0.04	-0.02	-0.23	-0.16	-0.06	0.10	0.08	-0.01	0.05	-0.02	0.19	0.34	0.27	0.20	0.15	0.02	0.12	0.21	-0.24	0.30	0.44	-0.02	0.31	-0.04		0.48	0.12
Cat	-0.08	-0.05	-0.04	0.03	-0.15	0.49	-0.19	-0.39	0.10	-0.33	0.00	0.42	0.06	-0.19	-0.18	-0.15	-0.16	-0.31	-0.01	-0.15	0.24	0.21	-0.11	0.01	0.10	0.13	0.20	0.12	0.03	0.27	0.01	0.19	0.18	-0.23	0.15	0.40	-0.15	0.31	0.10	0.48		0.04
Gpx1	0.73	0.67	0.70	0.68	0.57	-0.10	0.47	0.36	-0.24	0.19	0.56	0.06	0.64	0.60	0.30	0.36	0.18	0.32	0.33	0.42	-0.45	-0.22	0.64	0.62	0.60	0.58	0.63	0.50	0.57	-0.03	0.52	0.26	0.50	0.56	0.70	-0.04	0.50	0.34	-0.30	0.12	0.04	

Data analyzed were Log2(fold change)or −ΔΔCq calculated for all groups with CTL at L12 as reference group. Correlations were realized using Pearson's test. Results were coloured function of the strength of the correlation from white boxes for a negative correlation to dark green for strong positive correlation.

Figure S2: Pearson correlation table between all mammary gland genes expressions on data normalized by the period of lactation.

	Lpl	Fasn	Acaca	Acacb	Fabp3	Fabp4	Scd1	Dgat1	Dgat2	Plin2	Glut1	Pgm1	Ugp2	B4galt1	Lalba	Csn2	Wap	Slc7a5	Aqp1	Aqp3	Cpt1a	Acads	Pdha1	Cs	Mtco1	Atp5f1a	Srebf1	Pparg	Spot14	Lxra	Akt1	Mtor	Stat5	Insr	Prlr	Oxtr	Ghr	Igf1r	Esr1	Sod1	Cat	Gpx1
**Lpl**		0.78	0.89	0.72	0.58	0.58	0.53	0.11	-0.16	0.08	0.26	0.41	0.85	0.44	0.43	0.41	0.19	-0.05	0.34	0.32	0.44	0.57	0.58	0.60	0.50	0.57	0.58	0.61	0.66	0.55	0.57	0.54	0.64	0.50	0.75	0.60	0.59	0.35	0.36	0.24	0.08	0.53
**Fasn**	0.78		0.84	0.75	0.50	0.41	0.42	0.13	-0.17	0.13	0.19	0.38	0.72	0.42	0.54	0.58	0.27	0.04	0.47	0.44	0.34	0.55	0.50	0.54	0.49	0.44	0.44	0.44	0.72	0.51	0.51	0.51	0.65	0.49	0.69	0.56	0.46	0.35	0.59	0.22	0.14	0.33
**Acaca**	0.89	0.84		0.77	0.55	0.46	0.62	0.04	-0.25	0.07	0.28	0.41	0.81	0.55	0.47	0.47	0.22	-0.04	0.34	0.35	0.40	0.58	0.59	0.65	0.53	0.62	0.57	0.50	0.64	0.58	0.60	0.55	0.69	0.50	0.73	0.67	0.59	0.40	0.38	0.18	0.10	0.51
**Acacb**	0.72	0.75	0.77		0.20	0.54	0.39	-0.08	0.03	-0.05	0.07	0.39	0.64	0.15	0.39	0.39	0.18	-0.20	0.27	0.38	0.22	0.35	0.40	0.39	0.42	0.46	0.48	0.38	0.68	0.45	0.37	0.41	0.61	0.35	0.81	0.64	0.51	0.21	0.39	0.56	0.31	0.41
**Fabp3**	0.58	0.50	0.55	0.20		0.12	0.24	0.28	-0.60	0.45	0.36	0.18	0.67	0.73	0.75	0.68	0.57	0.61	0.45	0.17	0.42	0.65	0.49	0.55	0.37	0.40	0.16	0.41	0.34	0.48	0.63	0.55	0.46	0.52	0.23	0.45	0.33	0.45	0.50	-0.22	-0.22	-0.03
**Fabp4**	0.58	0.41	0.46	0.54	0.12		0.29	-0.15	0.01	-0.10	0.14	0.19	0.50	-0.17	0.16	0.18	-0.01	-0.29	0.34	0.17	0.32	0.27	0.36	0.37	0.44	0.41	0.51	0.71	0.43	0.45	0.33	0.44	0.41	0.14	0.62	0.63	0.64	0.26	0.28	0.52	0.30	0.29
**Scd1**	0.53	0.42	0.62	0.39	0.24	0.29		0.03	0.13	0.00	0.08	0.16	0.36	0.41	0.09	0.10	-0.07	-0.03	0.05	0.20	0.20	0.23	0.31	0.33	0.28	0.36	0.32	0.25	0.34	0.28	0.30	0.19	0.31	0.25	0.31	0.26	0.39	0.23	0.11	-0.11	-0.11	0.34
**Dgat1**	0.11	0.13	0.04	-0.08	0.28	-0.15	0.03		-0.08	0.77	-0.44	-0.32	0.00	0.42	0.40	0.43	0.35	0.46	0.44	0.42	0.13	0.20	0.16	0.15	0.29	-0.08	-0.28	0.02	0.08	0.07	0.30	0.13	0.14	0.49	-0.04	-0.18	-0.20	0.14	0.49	-0.50	-0.23	-0.38
**Dgat2**	-0.16	-0.17	-0.25	0.03	-0.60	0.01	0.13	-0.08		-0.24	-0.32	-0.08	-0.46	-0.33	-0.47	-0.49	-0.50	-0.32	-0.39	0.14	-0.04	-0.27	-0.35	-0.37	-0.24	-0.23	-0.20	-0.21	-0.09	-0.18	-0.45	-0.35	-0.27	-0.17	-0.13	-0.27	-0.04	-0.12	-0.36	0.21	0.08	0.10
**Plin2**	0.08	0.13	0.07	-0.05	0.45	-0.10	0.00	0.77	-0.24		-0.38	-0.25	0.15	0.53	0.65	0.67	0.58	0.53	0.58	0.35	0.20	0.33	0.36	0.29	0.36	0.11	-0.27	0.08	0.12	0.24	0.43	0.33	0.22	0.58	0.00	-0.08	-0.04	0.26	0.59	-0.45	-0.09	-0.48
**Glut1**	0.26	0.19	0.28	0.07	0.36	0.14	0.08	-0.44	-0.32	-0.38		0.37	0.27	0.12	0.12	0.06	0.14	0.13	-0.22	-0.20	0.06	0.08	-0.02	0.16	-0.16	0.09	0.14	0.21	-0.01	0.02	-0.09	0.02	-0.03	-0.22	0.01	0.32	0.17	0.03	-0.17	0.30	0.07	0.09
**Pgm1**	0.41	0.38	0.41	0.39	0.18	0.19	0.16	-0.32	-0.08	-0.25	0.37		0.49	0.11	0.11	0.12	0.02	-0.11	0.11	0.05	0.23	0.26	0.21	0.26	0.13	0.28	0.36	0.36	0.27	0.33	0.12	0.32	0.30	0.00	0.37	0.18	0.27	0.15	0.00	0.25	0.12	0.27
**Ugp2**	0.85	0.72	0.81	0.64	0.67	0.50	0.36	0.00	-0.46	0.15	0.27	0.49		0.46	0.56	0.55	0.37	0.03	0.47	0.30	0.49	0.67	0.66	0.66	0.57	0.68	0.66	0.66	0.71	0.66	0.70	0.70	0.76	0.50	0.76	0.63	0.61	0.45	0.41	0.20	0.05	0.40
**B4galt1**	0.44	0.42	0.55	0.15	0.73	-0.17	0.41	0.42	-0.33	0.53	0.12	0.11	0.46		0.55	0.53	0.42	0.61	0.30	0.31	0.39	0.59	0.47	0.54	0.36	0.34	-0.03	0.20	0.21	0.47	0.51	0.44	0.40	0.61	0.12	0.22	0.24	0.45	0.33	-0.44	-0.27	0.01
**Lalba**	0.43	0.54	0.47	0.39	0.75	0.16	0.09	0.40	-0.47	0.65	0.12	0.11	0.56	0.55		0.97	0.89	0.55	0.57	0.45	0.28	0.52	0.52	0.53	0.38	0.34	0.02	0.34	0.48	0.43	0.56	0.49	0.48	0.57	0.37	0.45	0.29	0.34	0.77	0.00	-0.04	-0.36
**Csn2**	0.41	0.58	0.47	0.39	0.68	0.18	0.10	0.43	-0.49	0.67	0.06	0.12	0.55	0.53	0.97		0.86	0.51	0.64	0.47	0.26	0.50	0.52	0.53	0.44	0.33	0.03	0.34	0.47	0.42	0.56	0.51	0.49	0.55	0.39	0.42	0.27	0.33	0.81	-0.03	0.00	-0.38
**Wap**	0.19	0.27	0.22	0.18	0.57	-0.01	-0.07	0.35	-0.50	0.58	0.14	0.02	0.37	0.42	0.89	0.86		0.50	0.42	0.37	0.16	0.33	0.38	0.37	0.19	0.19	-0.13	0.22	0.28	0.25	0.35	0.31	0.29	0.40	0.19	0.26	0.09	0.21	0.59	0.04	-0.07	-0.54
**Slc7a5**	-0.05	0.04	-0.04	-0.20	0.61	-0.29	-0.03	0.46	-0.32	0.53	0.13	-0.11	0.03	0.61	0.55	0.51	0.50		0.25	0.14	0.08	0.23	0.08	0.16	0.03	-0.11	-0.43	0.00	-0.08	0.12	0.19	0.15	0.00	0.28	-0.30	0.05	-0.05	0.22	0.39	-0.45	-0.32	-0.54
**Aqp1**	0.34	0.47	0.34	0.27	0.45	0.34	0.05	0.44	-0.39	0.58	-0.22	0.11	0.47	0.30	0.57	0.64	0.42	0.25		0.36	0.34	0.52	0.51	0.52	0.67	0.34	0.18	0.49	0.40	0.57	0.63	0.68	0.58	0.56	0.41	0.36	0.29	0.42	0.70	-0.15	0.24	-0.13
**Aqp3**	0.32	0.44	0.35	0.38	0.17	0.17	0.20	0.42	0.14	0.35	-0.20	0.05	0.30	0.31	0.45	0.47	0.37	0.14	0.36		0.59	0.51	0.46	0.52	0.48	0.41	0.14	0.36	0.52	0.52	0.45	0.41	0.56	0.65	0.41	0.35	0.43	0.60	0.44	0.13	0.12	-0.09
**Cpt1a**	0.44	0.34	0.40	0.22	0.42	0.32	0.20	0.13	-0.04	0.20	0.06	0.23	0.49	0.39	0.28	0.26	0.16	0.08	0.34	0.59		0.88	0.59	0.69	0.71	0.74	0.36	0.71	0.41	0.85	0.63	0.76	0.70	0.66	0.42	0.48	0.73	0.92	0.22	0.04	0.02	0.21
**Acads**	0.57	0.55	0.58	0.35	0.65	0.27	0.23	0.20	-0.27	0.33	0.08	0.26	0.67	0.59	0.52	0.50	0.33	0.23	0.52	0.51	0.88		0.73	0.81	0.76	0.81	0.42	0.68	0.60	0.92	0.82	0.88	0.85	0.80	0.56	0.58	0.74	0.88	0.44	-0.05	-0.01	0.24
**Pdha1**	0.58	0.50	0.59	0.40	0.49	0.36	0.31	0.16	-0.35	0.36	-0.02	0.21	0.66	0.47	0.52	0.52	0.38	0.08	0.51	0.46	0.59	0.73		0.90	0.69	0.84	0.50	0.64	0.67	0.74	0.83	0.80	0.77	0.83	0.69	0.55	0.68	0.63	0.60	-0.01	0.01	0.24
**Cs**	0.60	0.54	0.65	0.39	0.55	0.37	0.33	0.15	-0.37	0.29	0.16	0.26	0.66	0.54	0.53	0.53	0.37	0.16	0.52	0.52	0.69	0.81	0.90		0.76	0.84	0.51	0.70	0.59	0.81	0.84	0.82	0.82	0.80	0.66	0.64	0.74	0.74	0.56	-0.02	0.13	0.25
**Mtco1**	0.50	0.49	0.53	0.42	0.37	0.44	0.28	0.29	-0.24	0.36	-0.16	0.13	0.57	0.36	0.38	0.44	0.19	0.03	0.67	0.48	0.71	0.76	0.69	0.76		0.74	0.50	0.74	0.55	0.80	0.80	0.84	0.82	0.74	0.64	0.56	0.68	0.70	0.51	-0.09	0.27	0.25
**Atp5f1a**	0.57	0.44	0.62	0.46	0.40	0.41	0.36	-0.08	-0.23	0.11	0.09	0.28	0.68	0.34	0.34	0.33	0.19	-0.11	0.34	0.41	0.74	0.81	0.84	0.84	0.74		0.68	0.71	0.67	0.84	0.83	0.85	0.86	0.69	0.71	0.65	0.84	0.75	0.36	0.13	0.11	0.41
**Srebf1**	0.58	0.44	0.57	0.48	0.16	0.51	0.32	-0.28	-0.20	-0.27	0.14	0.36	0.66	-0.03	0.02	0.03	-0.13	-0.43	0.18	0.14	0.36	0.42	0.50	0.51	0.50	0.68		0.52	0.64	0.45	0.56	0.50	0.67	0.24	0.74	0.58	0.62	0.33	0.11	0.23	0.22	0.60
**Pparg**	0.61	0.44	0.50	0.38	0.41	0.71	0.25	0.02	-0.21	0.08	0.21	0.36	0.66	0.20	0.34	0.34	0.22	0.00	0.49	0.36	0.71	0.68	0.64	0.70	0.74	0.71	0.52		0.52	0.79	0.63	0.81	0.67	0.49	0.62	0.61	0.78	0.66	0.35	0.23	0.11	0.20
**Spot14**	0.66	0.72	0.64	0.68	0.34	0.43	0.34	0.08	-0.09	0.12	-0.01	0.27	0.71	0.21	0.48	0.47	0.28	-0.08	0.40	0.52	0.41	0.60	0.67	0.59	0.55	0.67	0.64	0.52		0.59	0.64	0.62	0.79	0.59	0.82	0.58	0.63	0.41	0.57	0.23	0.10	0.29
**Lxra**	0.55	0.51	0.58	0.45	0.48	0.45	0.28	0.07	-0.18	0.24	0.02	0.33	0.66	0.47	0.43	0.42	0.25	0.12	0.57	0.52	0.85	0.92	0.74	0.81	0.80	0.84	0.45	0.79	0.59		0.78	0.93	0.86	0.75	0.64	0.66	0.85	0.88	0.41	0.09	0.14	0.27
**Akt1**	0.57	0.51	0.60	0.37	0.63	0.33	0.30	0.30	-0.45	0.43	-0.09	0.12	0.70	0.51	0.56	0.56	0.35	0.19	0.63	0.45	0.63	0.82	0.83	0.84	0.80	0.83	0.56	0.63	0.64	0.78		0.86	0.87	0.81	0.62	0.60	0.65	0.71	0.63	-0.16	-0.02	0.22
**Mtor**	0.54	0.51	0.55	0.41	0.55	0.44	0.19	0.13	-0.35	0.33	0.02	0.32	0.70	0.44	0.49	0.51	0.31	0.15	0.68	0.41	0.76	0.88	0.80	0.82	0.84	0.85	0.50	0.81	0.62	0.93	0.86		0.87	0.75	0.64	0.64	0.76	0.79	0.54	0.00	0.11	0.18
**Stat5**	0.64	0.65	0.69	0.61	0.46	0.41	0.31	0.14	-0.27	0.22	-0.03	0.30	0.76	0.40	0.48	0.49	0.29	0.00	0.58	0.56	0.70	0.85	0.77	0.82	0.82	0.86	0.67	0.67	0.79	0.86	0.87	0.87		0.75	0.82	0.70	0.75	0.75	0.54	0.12	0.16	0.34
**Insr**	0.50	0.49	0.50	0.35	0.52	0.14	0.25	0.49	-0.17	0.58	-0.22	0.00	0.50	0.61	0.57	0.55	0.40	0.28	0.56	0.65	0.66	0.80	0.83	0.80	0.74	0.69	0.24	0.49	0.59	0.75	0.81	0.75	0.75		0.53	0.44	0.56	0.71	0.61	-0.18	0.08	0.11
**Prlr**	0.75	0.69	0.73	0.81	0.23	0.62	0.31	-0.04	-0.13	0.00	0.01	0.37	0.76	0.12	0.37	0.39	0.19	-0.30	0.41	0.41	0.42	0.56	0.69	0.66	0.64	0.71	0.74	0.62	0.82	0.64	0.62	0.64	0.82	0.53		0.70	0.68	0.37	0.46	0.42	0.31	0.49
**Oxtr**	0.60	0.56	0.67	0.64	0.45	0.63	0.26	-0.18	-0.27	-0.08	0.32	0.18	0.63	0.22	0.45	0.42	0.26	0.05	0.36	0.35	0.48	0.58	0.55	0.64	0.56	0.65	0.58	0.61	0.58	0.66	0.60	0.64	0.70	0.44	0.70		0.80	0.53	0.42	0.38	0.27	0.28
**Ghr**	0.59	0.46	0.59	0.51	0.33	0.64	0.39	-0.20	-0.04	-0.04	0.17	0.27	0.61	0.24	0.29	0.27	0.09	-0.05	0.29	0.43	0.73	0.74	0.68	0.74	0.68	0.84	0.62	0.78	0.63	0.85	0.65	0.76	0.75	0.56	0.68	0.80		0.76	0.27	0.26	0.23	0.39
**Igf1r**	0.35	0.35	0.40	0.21	0.45	0.26	0.23	0.14	-0.12	0.26	0.03	0.15	0.45	0.45	0.34	0.33	0.21	0.22	0.42	0.60	0.92	0.88	0.63	0.74	0.70	0.75	0.33	0.66	0.41	0.88	0.71	0.79	0.75	0.71	0.37	0.53	0.76		0.31	-0.04	0.02	0.12
**Esr1**	0.36	0.59	0.38	0.39	0.50	0.28	0.11	0.49	-0.36	0.59	-0.17	0.00	0.41	0.33	0.77	0.81	0.59	0.39	0.70	0.44	0.22	0.44	0.60	0.56	0.51	0.36	0.11	0.35	0.57	0.41	0.63	0.54	0.54	0.61	0.46	0.42	0.27	0.31		-0.07	0.04	-0.27
**Sod1**	0.24	0.22	0.18	0.56	-0.22	0.52	-0.11	-0.50	0.21	-0.45	0.30	0.25	0.20	-0.44	0.00	-0.03	0.04	-0.45	-0.15	0.13	0.04	-0.05	-0.01	-0.02	-0.09	0.13	0.23	0.23	0.23	0.09	-0.16	0.00	0.12	-0.18	0.42	0.38	0.26	-0.04	-0.07		0.35	0.23
**Cat**	0.08	0.14	0.10	0.31	-0.22	0.30	-0.11	-0.23	0.08	-0.09	0.07	0.12	0.05	-0.27	-0.04	0.00	-0.07	-0.32	0.24	0.12	0.02	-0.01	0.01	0.13	0.27	0.11	0.22	0.11	0.10	0.14	-0.02	0.11	0.16	0.08	0.31	0.27	0.23	0.02	0.04	0.35		0.17
**Gpx1**	0.53	0.33	0.51	0.41	-0.03	0.29	0.34	-0.38	0.10	-0.48	0.09	0.27	0.40	0.01	-0.36	-0.38	-0.54	-0.54	-0.13	-0.09	0.21	0.24	0.24	0.25	0.25	0.41	0.60	0.20	0.29	0.27	0.22	0.18	0.34	0.11	0.49	0.28	0.39	0.12	-0.27	0.23	0.17	

Data analyzed were Log2(fold change)or –ΔΔCq calculated for each period with the CTL of the day as reference group. Correlations were realized using Pearson's test. Results were coloured function of the strength of the correlation from white boxes for a negative correlation to dark green for strong positive correlation.

Figure S3 : PLS-DA score plot on non-normalized mammary gene expression separating groups according to the diet and the lactation period.

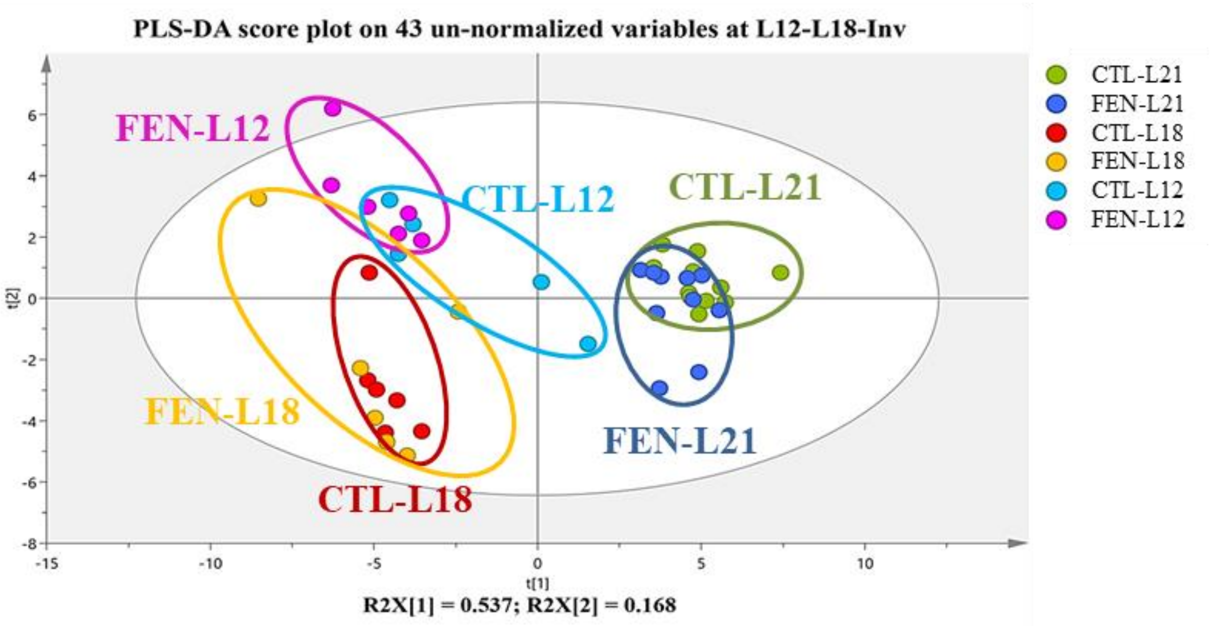

The **PLS-DA model** presented a cumulative modeled variation (R2X) value of 71% and various predictive abilities for each [diet-lactation time] group according to cumulative variation (R2Y) and predicted variation (Q2Y) values *i.e* CTL – L21 : R2(Y) cum = 0.39, Q2 cum = 0.38 ; FEN – L21 : R2(Y) cum = 0.24, Q2 cum = 0.22 ; CTL-L18 : R2(Y) cum = 0.35, Q2 cum = 0.33 ; FEN–L18 : R2(Y) cum = 0.30, Q2 cum = 0.21 ; CTL–L12 : R2(Y) cum = 0.06, Q2 cum = -0.02 ; FEN–L12 : R2(Y) cum = 0.45, Q2 cum = 0.38. Only one sample (FEN-L12) was identified as outlier according to the Hotelling's T2 grey circle, corresponding to a multivariate generalization of the 95% confidence interval.

Figure S4: Food intake of dams supplemented or not with 1g/kg/day fenugreek during lactation

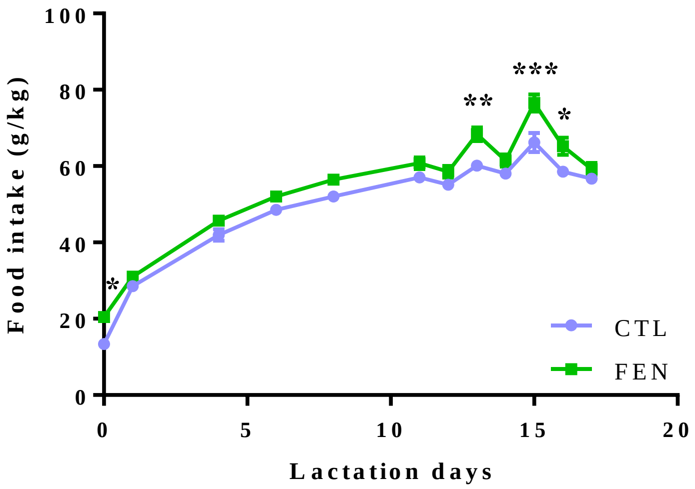

Values are mean ± SEM and n= 16-17 per group and per day. Data were analyzed with Two-way ANOVA with repeated measures for day effect followed by Sidak *post-hoc* test for diet effect. ***, **** and ******* p <0.05, p <0.01 andp <0.01 for diet comparison.

Table S1: Validation of RNA extraction in quantity and quality and of primer design in efficiency and specificity.

Tissues	RNA extraction			Primer design		
	RNA concentration (µg/ mL)	Ratio 260/280 nm	Ratio 260/230 nm	Amplicon size (bp)	Efficiency (%)	$\Delta Cq_{(RT-)}$
**MG**	593 ± 268	2.01 ± 0.02	2.25 ± 0.08	124.7 ± 28.6	94.8 ± 4.5	21.0 ± 5.0
**PG**	627 ± 179	2.03 ± 0.01	2.20 ± 0.11		95.2 ± 5.6	16.8 ± 4.7

Values are mean ± standard deviation. MG and PG: mammary and pituitary glands, respectively.

$\Delta Cq_{(RT-)}$ = mean $Cq_{(RT-)}$ – mean $Cq_{(sample)}$ with a value of 50 assigned for unamplified samples for calculation.
